# Progressive striatonigral degeneration in a transgenic mouse model of multiple system atrophy: translational implications for interventional therapies

**DOI:** 10.1186/s40478-017-0504-y

**Published:** 2018-01-03

**Authors:** Violetta Refolo, Francesco Bez, Alexia Polissidis, Daniela Kuzdas-Wood, Edith Sturm, Martina Kamaratou, Werner Poewe, Leonidas Stefanis, M. Angela Cenci, Marina Romero-Ramos, Gregor K. Wenning, Nadia Stefanova

**Affiliations:** 1 Division of Neurobiology, Department of Neurology, Medical University of Innsbruck, Innrain 66 / G2, 6020 Innsbruck, Austria; 2 Basal Ganglia Pathophysiology Lab, Lund University, Lund, Sweden; 3 Center of Clinical, Experimental Surgery and Translational Research, Biomedical Research Foundation of the Academy of Athens, Athens, Greece; 4 Second Department of Neurology, National and Kapodistrian University of Athens Medical School, Athens, Greece; 5 Department of Biomedicine, Aarhus University, Aarhus, Denmark

**Keywords:** Multiple system atrophy, Striatonigral degeneration, α-synuclein, transgenic mouse, Neuroinflammation

## Abstract

Multiple system atrophy (MSA) is a rapidly progressive neurodegenerative disorder characterized by widespread oligodendroglial cytoplasmic inclusions of filamentous α-synuclein, and neuronal loss in autonomic centres, basal ganglia and cerebellar circuits. It has been suggested that primary oligodendroglial α-synucleinopathy may represent a trigger in the pathogenesis of MSA, but the mechanisms underlying selective vulnerability and disease progression are unclear. The post-mortem analysis of MSA brains provides a static final picture of the disease neuropathology, but gives no clear indication on the sequence of pathogenic events in MSA. Therefore, alternative methods are needed to address these issues. We investigated selective vulnerability and disease progression in the transgenic PLP-α-syn mouse model of MSA characterized by targeted oligodendroglial α-synuclein overexpression aiming to provide a neuropathological correlate of motor deterioration. We show progressive motor deficits that emerge at 6 months of age and deteriorate up to 18 months of follow-up. The motor phenotype was associated with dopaminergic cell loss in the substantia nigra pars compacta at 6 months, followed by loss of striatal dopaminergic terminals and DARPP32-positive medium sized projection neurons at 12 months. Olivopontocerebellar motor loops remained spared in the PLP-α-syn model of MSA. These findings replicate progressive striatonigral degeneration underlying Parkinson-variant MSA. The initiation of the degenerative process was linked to an increase of soluble oligomeric α-synuclein species between 2 and 6 months. Early region-specific α-synuclein-associated activation profile of microglia was found in MSA substantia nigra. The role of abnormal neuroinflammatory signalling in disease progression was further supported by increased levels of CD68, CCL3, CCL5 and M-CSF with a peak in aged PLP-α-syn mice. In summary, transgenic PLP-α-syn mice show a distinctive oligodendroglial α-synucleinopathy that is associated with progressive striatonigral degeneration linked to abnormal neuroinflammatory response. The model provides a relevant tool for preclinical therapeutic target discovery for human Parkinson-variant MSA.

**Electronic supplementary material:**

The online version of this article (10.1186/s40478-017-0504-y) contains supplementary material, which is available to authorized users.

## Introduction

Multiple system atrophy (MSA) is a fatal rapidly progressive neurodegenerative disorder without effective therapy [13]. In the Western hemisphere the Parkinson variant predominates over the cerebellar subtype of MSA in up to 4:1 of the cases [13]. The clinical diagnosis of MSA relies on the coexistence of autonomic failure and parkinsonian or cerebellar motor impairment, however, a definite diagnosis still requires autopsy [23]. Autonomic or other non-motor features such as urogenital disturbances, neurogenic orthostatic hypotension or REM sleep behaviour disorder may precede the onset of the motor syndrome [30].

The pathological hallmark feature of MSA is the presence of glial cytoplasmic inclusions (GCIs) in oligodendrocytes [42]. The major component of the GCIs is filamentous α-synuclein (α-syn) [31, 53, 63, 67]. In addition, neuronal α-syn aggregates have also been reported in MSA [10]. Analysis of SNCA mRNA identifies increased levels of SNCA140 (full-length α-syn) and SNCA112 isoform in substantia nigra, striatum, cerebellar cortex and nucleus dentatus of MSA patients as compared to healthy controls and PD patients, while SNCA126 mRNA is significantly lower in MSA cases [8]. Epitope-specific antibodies confirm the over-expression of full-length α-syn and α-syn 112 isoform (detectable by 5G4 antibody, which is considered to bind predominantly to aggregate α-syn [8, 33]. The end-stage neuropathological features of MSA include striatonigral degeneration (SND) and olivopontocerebellar atrophy (OPCA), as well as neuronal loss in brainstem and spinal cord autonomic centres [5, 45, 69, 70]. Despite the fact that the major focus of α-syn accumulation are the oligodendrocytes in MSA, only minor changes of oligodendroglial numbers (mostly non-significant) are detectable by stereological analysis of MSA brains [41, 45, 46]. In mildly or moderately affected MSA brains no significant demyelination is detected [28]. A region-specific increase in astrocyte numbers (astrogliosis) is found in basal ganglia and cortex, but not in white matter of end-stage MSA [41, 45, 46]. Finally, widespread microgliosis has been reported in MSA brains in parallel to the α-syn pathology [28, 29, 41, 45, 46].

It has been suggested that primary oligodendroglial α-synucleinopathy may represent a trigger in the pathogenesis of MSA [71]. However, the mechanisms underlying selective vulnerability and disease progression are unclear. The post-mortem analysis of MSA brains provides a static final picture of the disease neuropathology, but gives no clear indication on the sequence of pathogenic events in MSA. Therefore, alternative methods are needed to address selective vulnerability and disease progression in MSA.

Several rodent models have been developed over the last two decades to serve as pre-clinical testbeds of MSA [59, 60, 64]. Each of these models shows advantages and limitations as previously discussed [60]. The transgenic mouse overexpressing α-syn in oligodendroglia under the proteolipid protein (PLP) promoter [32] is especially interesting because this transgenic murine model of MSA develops a progressive phenotype, including non-motor and motor symptoms similar to the human disease [7, 18, 19, 26, 34, 35, 57, 58, 61]. However, studies on the progressive evolution of the MSA-like phenotype from the very early stages are limited in the PLP-α-syn mouse. We here investigate motor dysfunction and neurodegeneration in aging PLP-α-syn mice in order to identify patterns of selective vulnerability downstream of GCI pathology. Our data provide new insights into MSA pathogenesis and they are therefore important for future preclinical studies investigating candidate neuroprotective agents for the human disease.

## Materials and methods

### Experimental design

Homozygous transgenic PLP-α-syn mice (MGI:3,604,008) overexpressing human α-syn under the PLP-promoter [32] and background-, age- and sex-matched non-transgenic C57Bl/6 mice were used in this study. They were bred and housed in a temperature-controlled room under a 12/12 h dark/light cycle, with free access to food and water, in the animal facility of the Medical University of Innsbruck, under special pathogen free conditions. During the study, all efforts were made to minimize the number of animals used and their suffering. All experiments were performed in accordance with the Austrian law and after permission for animal experiments by the authorities (BMWF-66.011/0034-II/10b/2010 and BMWFW-66.011/0041-WF/II/3b/2014).

### Motor analysis

Motor behaviour in PLP-α-syn mice and non-transgenic controls was tested at 2, 6, 12 and 18 months of age by applying the beam walking test, the pole test, gait analysis, and grip strength analysis.

#### Beam walking

Motor coordination and balance was assessed with the method adapted from Fernagut et al. [17] by measuring the ability of the mice to traverse a narrow beam to reach a dark goal box. The beams consist of two different strips of wood (each 50 cm long; one was 1.6 cm and the other 0.9 cm square cross-section) placed horizontally 20 and 50 cm above the floor, respectively. During training, three daily sessions of three trials (nine crossings) were performed using the 1.6 cm square large beam. Mice were then tested using the 0.9 cm square beam. Mice were allowed to perform three consecutive trials. The time to cross the beam and the number of sideslips (errors) were recorded on each trial, and the mean number of sideslips during a three-trial session, as well as the best time, was kept as the variable.

#### Pole test

The pole test was performed according to established protocols [57]. Each mouse was habituated to the test the day before. A wooden vertical pole with rough surface, 1 cm wide and 50 cm high was used. Each mouse was placed with the head up at the top of the pole and the time for turning downwards (T_turn_) as well as the total time for climbing down the pole until the mouse reached the floor with the four paws (T_total_) was taken in 5 trials. The best performance of all the five trials was kept for the statistical analysis.

#### DigiGait test

Gait analysis was quantified with the DigiGait™ Imaging system (Mouse Specifics Inc., Boston, MA). Briefly, a video camera mounted below a transparent treadmill belt captured ventral images of each mouse. The images were automatically digitized and software algorithms analysed the digital images to define the area of each paw, and generate a set of periodic waveforms that describe the advance and retreat of the four limbs relative to the treadmill belt through consecutive strides. The software identified the portions of the paw that were in contact with the treadmill belt in the stance and swing phase of the stride, and measured several postural and kinematic metrics of gait dynamics. A treadmill physiological speed of 25 cm/s was applied and a maximum of 3 min was videotaped at constant light and camera settings.

#### Grip strength

Grip strength was measured by testing the ability of each mouse to remain clinging to an inverted cage lid at a height of 70 cm above the surface for a period of up to 1 min.

### CSF sampling

Mice received an overdose of thiopental. The head and neck were fixed and the muscles over cisterna magna were carefully removed. Blood was completely removed before puncture of the cisterna magna. A pulled capillary was used to puncture cisterna magna and collect CSF (up to 5 μl per mouse). CSF was stored at −80 °C before further analysis.

### Western blot analysis

#### Sequential α-syn extraction and quantification

Hemibrain tissue was collected after PBS perfusion and stored at −80 °C until analysis. We followed a previously published protocol with minor modifications [74]. Shortly, tissue was homogenized in Triton-X (TX) extraction buffer (50 mM Tris-base pH 7.6, 150 mM NaCl, 1% Triton-X-100, 2 mM EDTA) containing protease and phosphatase inhibitors. The lysate was sonicated and then centrifuged (120,000×g for 60 min at 4 °C) and the supernatant was collected (TX soluble fraction). The pellet was then washed 3 times with 1 M PBS/1% TX, centrifuged (13,000 x g for 15 min) and re-suspended in SDS extraction buffer (50 mM Tris pH 7.6, 150 mM NaCl, 1% Triton-X-100, 0.5% Na-deoxycholate, 1% SDS), sonicated, and centrifuged (120,000×g for 60 min at 4 °C) and the supernatant was collected (TX insoluble fraction). The samples were run on 12% SDS-PAGE gels. Primary antibodies included antibodies against α-syn [rabbit monoclonal anti-phospho-α-syn (pS-129), (1:500; Abcam, EP1536Y), human-specific monoclonal 4B12, (1:1000; Genetex, GTX21904), monoclonal syn-1, (1:1000; BD Biosciences, 619,787), polyclonal C20, (1:1000; Santa Cruz, sc-7011-R)] and β-actin (1:2000; Cell Signalling Technology, 8H10D10) as a loading control. The intensity of the immunoreactive bands was estimated by densitometric quantification using ImageJ (relative density, RD) and then normalized to the corresponding β-actin levels.

#### Subregion-specific α-syn analysis

Tissue homogenates from all selected brain regions were prepared in RIPA-lysis buffer containing 65 mM Tris-base, 150 mM NaCl, 1% Triton-X, 0.25% sodium deoxycholate, 1 mM EDTA, and a mix of phosphatase and protease inhibitors (“phosSTOP” and “Complete, mini, EDTA-free,” Roche Applied Science). BCA Protein Assay Kit (Pierce #23225, ThermoScientific) was used for determining the protein concentration. Equal amounts of protein (typically 20 μg) per sample were loaded on a 10% SDS-polyacrylamide gel for separation and then transferred on a polyvinyldifluoride membrane. Membranes were incubated in blocking buffer (20 mM Tris, 136 mM NaCl, pH 7.6, 0,1% Tween 20, 5% non-fat dry milk) and thereafter incubated overnight at 4 °C using one of the following primary antibodies: rabbit polyclonal anti GDNF (1:1000; Santa Cruz Biotechnologies, CA); rabbit polyclonal anti-BDNF (1:1000; Santa Cruz Biotechnologies, CA), mouse anti-mouse and human SNCA monoclonal antibody clone 42 (1:500; BD Transduction Laboratories, CA, USA), mouse anti-myelin basic protein (MBP, Abcam, ab62631), rabbit anti-p25α ([52], generously provided by Prof. Paul Henning Jensen, Aarhus University, Denmark), and mouse anti-myelin oligodendrocyte glycoprotein (MOG, clone 8-18C5 [43], generously provided by Prof. Markus Reindl, Medical University of Innsbruck, Austria).

After appropriate washing steps, membranes were incubated with HRP-linked secondary antibodies: anti-biotin, HRP-linked antibody (1:2500; Cell Signaling Technology, #7075); anti-rabbit IgG, HRP-linked (1:5000; Cell Signaling Technology, #7074); anti-mouse IgG, HRP-linked (1:4000; Cell Signaling Technology, #7076). Signals were visualized using a chemiluminescence ECL kit (Pierce, #32106, Thermo Scientific) and images were acquired using a CCD camera (LAS1000 system, Fuji Films). Optical density was measured on specific immunoreactive bands using ImageJ Software. Membranes were then stripped and re-probed with β-actin (1:120,000; Sigma-Aldrich) or mouse anti-alpha-tubulin (ab7750, Abcam). The optical density of specific bands was then normalized to the corresponding β-actin or alpha-tubulin levels.

### α-Syn ELISA

Selected brain areas were mixed 1:5 with RIPA buffer as described above, homogenized with IKA Ultra Turrax T8 and centrifuge for 15 min at 16000 g at 4 °C, the supernatant was collected and stored at −80 °C for further analysis. A commercially available ELISA kit for detection of α-syn (Invitrogen, CA, US) was applied to measure the concentration of α-syn in selected brain regions according to the manufacturer’s protocol.

The total level of α-syn in cerebrospinal fluid (CSF) was measured with an in-house ultra-sensitive sandwich ELISA for total α-syn as previously described (Emmanouilidou et al. 2011) adapted for measurement of mouse CSF. 3–5 μl samples of CSF were pooled from 3 to 6 animals of each group (control or MSA at 2, 6 or 18 months of age) or recombinant α-syn (as standard). The samples were controlled for haemoglobin content by absorbance at 414 nm, thereafter diluted in TBST/BSA (10 mM Tris-Cl, pH 7.6, 100 mM NaCl, 0.1% Tween-20 and 1% BSA), and measured in duplicate for α-syn levels. The limit of detection of this assay is 0.024 ng/ml. The total number of pooled samples per group that were detectable was 7/13 control and 6/11 MSA samples.

### Histopathology

Mice underwent transcardial perfusion with PBS followed by 4% paraformaldehyde for 15 min under deep thiopental anaesthesia. The brains were removed and post-fixed in the same fixative overnight at 4 °C. After 30% sucrose cryoprotection the brains were frozen and stored at −80 °C until further processing. Six series of 40 μm were cut on a cryotome (Leica, Nussloch, Germany). One series was directly mounted on slides and stained with cresyl violet. Immunohistochemistry on free-floating sections was performed using standard protocols and the following antibodies: anti-tyrosine hydroxylase (TH, 1:750, AB152, Millipore, Germany), anti-dopamine and cyclic AMP-regulated phosphoprotein (DARPP32, 1:15,000, 611,520, BD Transduction Laboratories), anti-Iba1 (1:800, ab108539, Abcam, UK), anti-CD68 (1:1000, MCA1957GA, Serotec (Bio-Rad), UK), anti-mouse MHCII (1:100, 14–5321, eBiosciences), anti-GFAP (1:1000, MAB3402, Millipore, Germany), rat anti-human α-synuclein (1:200, 15G7, Enzo Life Sciences, Switzerland), anti-phosphorylated α-synuclein (1:1000, phospho-S129, ab51253, Abcam, UK), anti-aggregated α-synuclein (1:1000, 5G4, Linaris, Germany), biotinylated anti-mouse, anti-rat and anti-rabbit IgG (1:500, respectively, BA-1000, BA-9400 and BA-2001, Vector Laboratories, US). The reaction was enhanced with ABC Elite kit (PK-6100, Vector Laboratories, US) and visualized with 3, 3′-diaminobenzidine. Cresyl violet was used for counterstaining of sections immunostained with anti-Iba1 antibody.

### Stereology and image analysis

Stereological estimate of number of neurons and microglia in striatonigral and olivopontocerebellar regions was performed by the use of the optical fractionator method with the StereoInvestigator Software (MicroBrightField) provided to a Nikon Eclipse 80i microscope with motorized stage and high resolution digital camera. A low power objective lens (2×, SPlan) was used to delineate the borders of the areas of interest at all levels in the rostro-caudal axis, according to anatomical points following the mouse brain atlas, between levels AP: +1.7 - -2.0 mm for striatum, −2.5 - -3.9 mm for SN, −3.8 - -4.6 mm for pontine nuclei and −6.7 - -7.5 mm for the inferior olives [20]. The described above cutting protocol and collection of section series yielded the following average number of sections per area: 15 for Striatum, 5 for SN, 4 for pontine nuclei and 4 for the inferior olives. The following design of the optical fractionator was used for the analyses. The cutting thickness of the sections was 40 μm, the final mounted thickness of the sections was 25 μm. Guard zones were 2.5 μm on each surface of the section, therefore resulting in a dissector height of 20 μm. The sampling frequency was chosen by adjusting the X-Y grid size between 80 μm and 250 μm (depending on the region), so that about 100–200 cells were counted in each structure. Counting frames of 50x50μm^2^ were systemically and randomly positioned throughout the region of interest led by the sampling grid as provided by the StereoInvestigator system. Actual counting was done using a 100× objective (NA 0.75). The coefficient of error (CE) did not exceed 0.07 and ratio CE2/CV2 was less than 0.5 as previously recommended [51].

The density of Purkinje cells in the cerebellar cortex was estimated by a previously described procedure [22, 57] by outlining a region to include only the Purkinje cell layer.

Astrogliosis was assessed by measuring the optical density (OD) of GFAP immunostaining as previously reported [55].

### Confocal microscopy

Three-dimensional stacks were acquired with an SP8 confocal microscope (Leica Microsystems, Wetzlar, Germany) using a HC PL APO CS2 63×, 1.3 NA glycerol immersion objective. Imaging was performed using WLL laser with excitation lines for Alexa 488 at 498 nm and for Alexa 594 at 590 nm. Fluorescence emission was detected in sequence 1 from 503 to 576 nm (Alexa 488) and in sequence 2 from 594 to 742 nm (Alexa 594). Images were acquired using the Leica LAS X 3.1.1 acquisition software (Leica Microsystems). Image deconvolution was performed using Huygens Professional software (Scientific Volume Imaging, Hilversum, Netherlands). The Ortho Slicer function was used to define spatial co-localization of signals in 3D.

### Characterization of microglia activation

Microglia cells were identified as a nucleus covered and surrounded by Iba1 immunostaining. During the stereological quantification, each microglial cell underwent morphological characterization, and was classified to one of four activation stages (namely A, B, C and D) based on their morphology (detailed description is provided in the results section). The quantification and morphological characterization was performed by a researcher blinded to the genotype and age of the animals.

### Cytokine/chemokine levels

Hemibrain tissue (*n* = 4 per group) was snap-frozen after PBS perfusion and stored at −80 °C until analysis. Tissue homogenates from hemibrains were prepared in RIPA-lysis buffer containing 65 mM Tris-base, 150 mM NaCl, 1% Triton-X, 0.25% sodium deoxycholate, 1 mM EDTA, and a mix of phosphatase and protease inhibitors (“phosSTOP” and “Complete, mini, EDTA-free,” Roche Applied Science). BCA Protein Assay Kit (Pierce #23225, ThermoScientific, Waltham, MA USA) was used for determining the total protein concentration. To measure the concentration of cytokines we used the ProcartaPlex® Multiplex Immunoassay (eBioscience, Waltham, MA USA) that uses the Luminex technology (multi-analyte profiling beads) to enable the simultaneous detection and quantitation of multiple cytokines and chemokines per sample, including IFN gamma; IL-12p70; IL-13; IL-1 beta; IL-2; IL-4; IL-5; IL-6; TNF alpha; GM-CSF; IL-18; IL-10; IL-17A; IL-22; IL-23; IL-27; IL-9; GRO alpha; IP-10; MCP-1; MCP-3; MIP-1 alpha; MIP-1 beta; MIP-2; RANTES; Eotaxin; IFN alpha; IL-15/IL-15R; IL-28; IL-31; IL-1 alpha; IL-3; G-CSF; LIF; ENA-78/CXCL5; and M-CSF. All samples were measured in duplicate and the mean values of the two reads were calculated and used in subsequent statistical analysis. Data are presented as pg cytokine/chemokine per mg total protein for the brain lysates.

### Statistical analysis

All data are presented as mean ± SEM. Two-way ANOVA (two-tailed) was used to compare groups with variables “genotype” and “age” or “genotype” and “brain region”, if not indicated otherwise. Bonferroni test was used to correct for multiple comparisons. Statistical significance was set at *p* < 0.05. Precise statistical data are reported in the Figure legends.

## Results

### α-Synuclein pathology and aging of PLP-α-syn mice

**Fig. 1:**
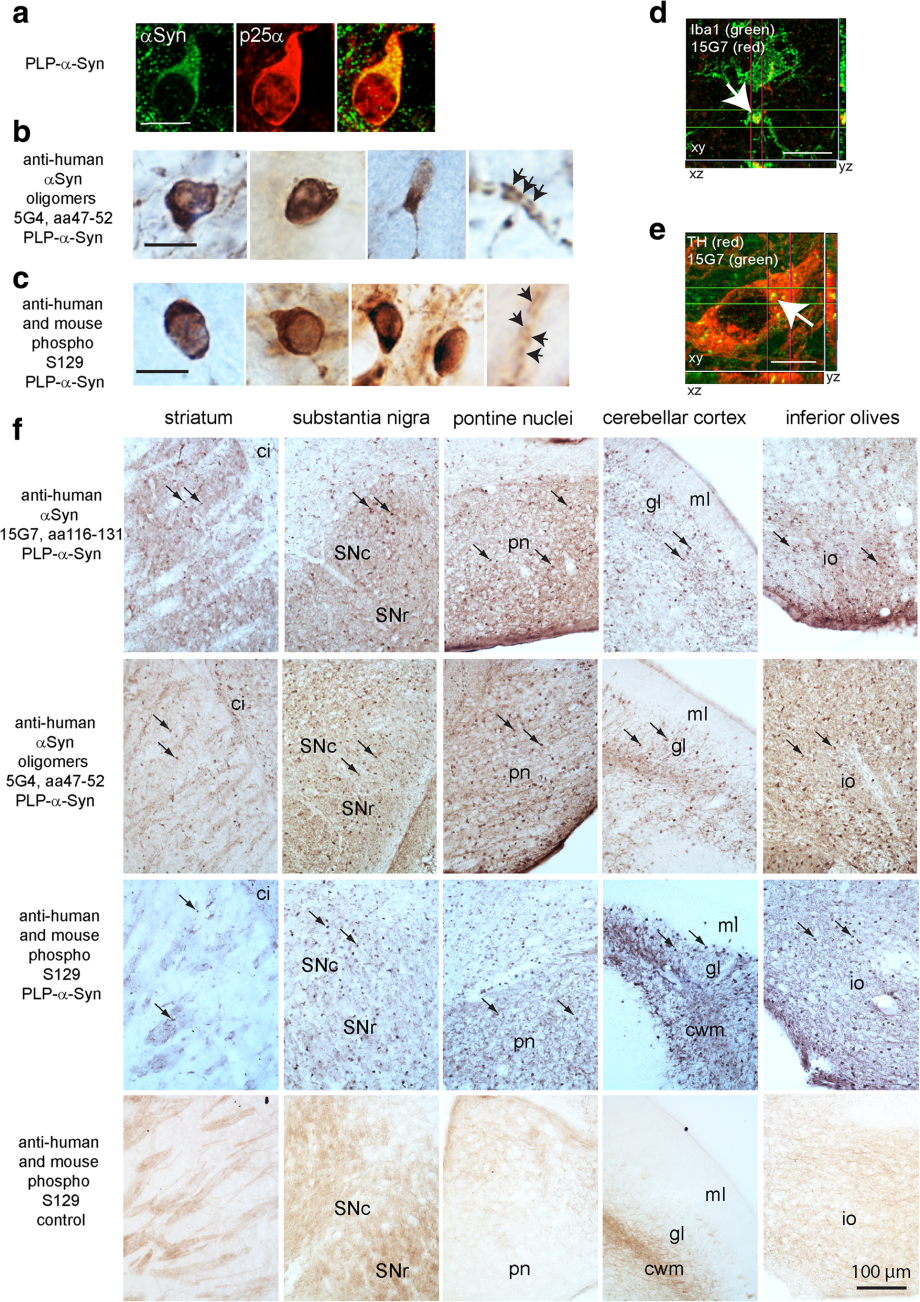
Synuclein neuropathology in the MSA mice. **a** Confocal images of immunofliorescence for human α-syn, detected with 15G7 antibody (green), and p25α (oligodendroglial marker, red), demonstrate the oligodendrocytic localization of α-syn. Human α-syn staining in oligodendrocytes is represented not only as diffuse cytoplasmic signal (due to the transgenic overexpression) but more commonly by dot-like-profiles that fuse into larger GCI-like aggregates in the cytoplasm of oligodendrocytes of the PLP-α-syn mice. These dense structures can be observed also by conventional light microscopy and immunohistochemistry applying antibodies specific for pathological α-syn species: (**b**) 5G4 anti-human α-syn oligomers, and (**c**) anti-phosphorylated S129 α- syn (human and mouse) antibodies demonstrate the morphology of GCIs in the oligodendrocytic cytoplasm (arrowheads) or within the myelin sheaths (arrows). Apart from the presence of human α-syn in the oligodendrocytes and myelin sheaths, we were able by confocal microscopy to identify single dot-like profiles (arrow) in microglial cells (**d**) and neurons (**e**), however no fusion of these profiles into larger aggregates was seen in microglia or neurons as compared to oligodendroglia, suggesting an effective degradation of the human α-syn uptaken from the extracellular space into these cells. **f** Microphotographs show a wide-spread distribution of α-syn GCI-like aggregates detected by the above mentioned antibodies in various brain areas (dense profiles indicated with arrows), always visible in the PLP-α-syn mice, but not in the wildtype controls. Abbreviations: ci, capsula interna; SNc, substantia nigra pars compacta; SNr, substantia nigra pars reticulata; pn, pontine nuclei; gl, granular layer; ml, molecular layer; cwm, cerebellar white matter; io, inferior olives. Scale bars, if not otherwise indicated, 5 μm

In the transgenic mice (hereafter named PLP-α-syn or MSA mice) the overexpression of human wild-type α-syn in oligodendrocytes under the PLP promoter resulted in a constitutive expression of the protein in oligodendrocytes, as confirmed by p25α co-expression throughout the brain (Fig. 1a). The oligodendroglial GCI-like pathology was detectable with specific antibodies targeting aggregated α-syn species [5G4 antibody as previously described [8, 33], Fig. 1b] and phosphorylated α-syn (pS129) [considered a dominant α-syn post-translational modification linked to lesions in α-synucleinopathies [1, 21], Fig. 1c]. Single Iba-1-positive microglial cells were found to contain small dot-like intracytoplasmic structures (<1 μm in diameter) expressing human α-syn (Fig. 1d), but those never accumulated into larger GCI-like inclusions (>2 μm in diameter), as demonstrated in oligodendrocytes (Fig. 1a-c). Similarly, single neuronal (Fig. 1e) and astroglial cells (not shown) were positive for human α-syn, but again the α-syn-positive profiles did not exceed 1 μm in diameter.

**Fig. 2:**
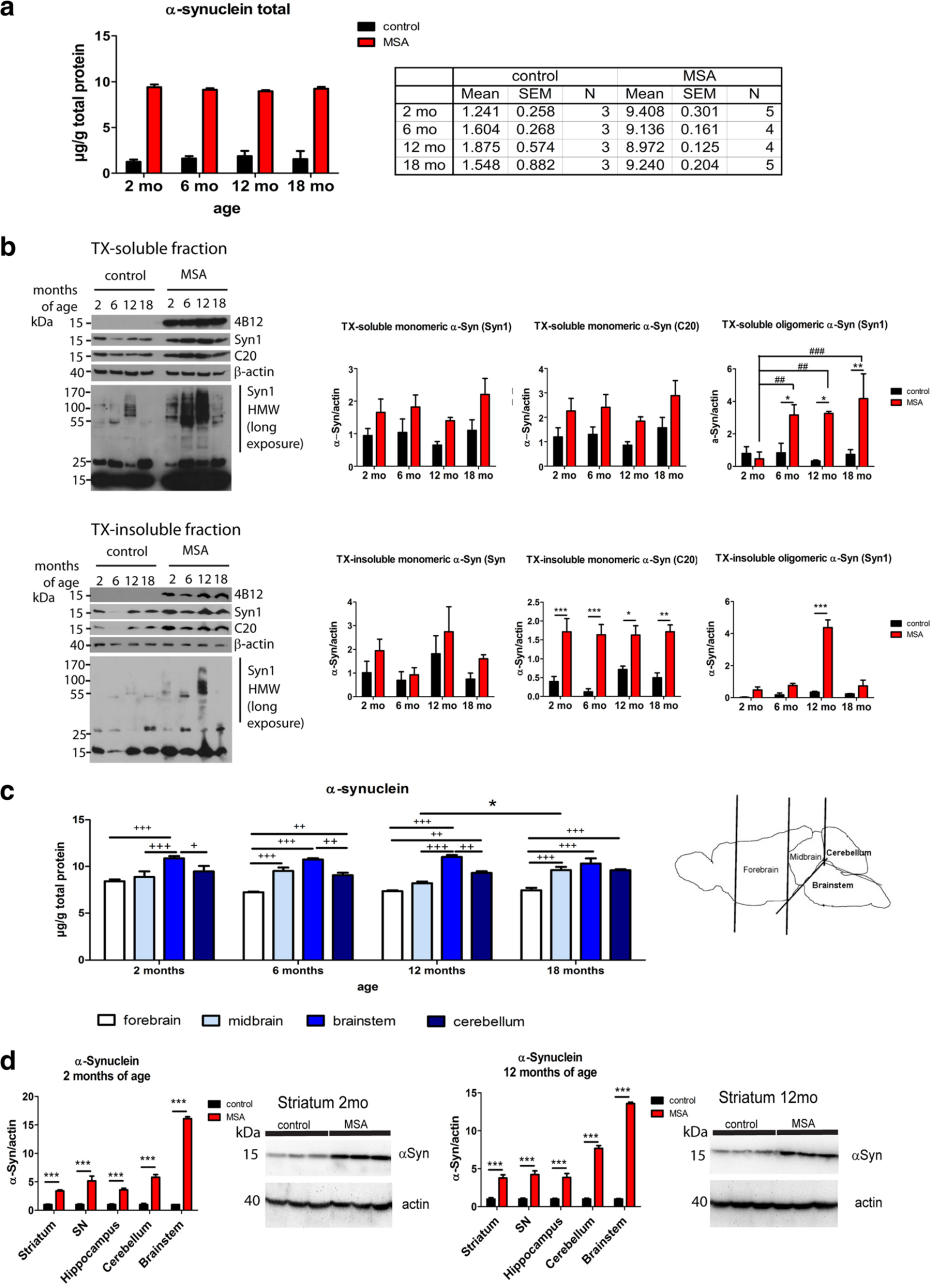
Biochemical analysis of α-synuclein expression in MSA transgenic mice during aging. **a** ELISA measurement of α-syn in whole brains shows 5 to 8 times higher α-syn levels in MSA than in control mice, with no significant changes over time in either group. **b** Subcellular fractionation of hemi-brains and subsequent Western immunoblotting using either the C20 polyclonal or the Syn1 monoconal Ab (*n* = 4 in each group) reveals higher α-syn levels in the TX-soluble fraction in MSA vs. control mice (two-way ANOVA with factors genotype and age: Syn1: genotype F_1,24_ = 12.58, *p* < 0.0016, age F_3,24_ = 1.244, *p* > 0.05; C20: genotype F_1,24_ = 14.38, *p* < 0.0009, age F_3,24_ = 1.505, p > 0.05); however, sub-group (age) differences are not significant following post-hoc Bonferroni test. Oligomeric species expression, assessed with the Syn1 Ab, tended to increase from 6 months onwards (Syn1: genotype F_1,24_ = 11.36, *p* < 0.0026, age F_3,24_ = 1.908, p > 0.05) without significant sub-group differences. SDS-soluble fractions reveal consistent increased monomeric α-syn levels regardless of age (C20: 2, 6, 12, 18 months: p < 0.001; 0.001; 0.05; 0.001) and HMW oligomeric species expression (again assessed with the Syn1 Ab) that peaked at 12 months (p < 0.001). The asterisk denotes a non-specific band at 25 kDA that is not included in the quantification of HMW species. **c** Subregional analysis by ELISA of MSA mouse brains (n = 4–5 for all groups) reveals an increase in α-syn levels with time only in the midbrain, from 12 to 18 months of age; the lowest α-syn levels are seen in the forebrain, while the highest appear in the lower brainstem (two-way ANOVA with factors region and age: effect of region F_3,56_ = 53.86, *p* < 0.0001, effect of age F_3,56_ = 1.104, *p* = 0.3552, interaction F_9,56_ = 2.042, *p* = 0.0512). (+ *p* < 0.05, ++ *p* < 0.01, +++ p < 0.001, * p < 0.05). On the side – representation of the dissection protocol of the subregions. **d** Western blot analysis confirms significantly higher α-syn expression in the MSA than in the control wildtype mice in all the considered areas at 2 and 12 months of age (*n* = 6 for each group)

GCI-like structures were found with variable density and distribution throughout the brain of PLP-α-syn mice (Fig. 1f). We found no overall change in the expression level of α-syn with aging in either control or PLP-α-syn mice, which had a 6.02 ± 0.58 fold increase in the α-syn expression level compared to the endogenous murine α-syn in wild-type control (Fig. 2a). In pooled CSF samples of control or PLP-α-syn mice at 2, 6 and 18 months of age no haemoglobin contamination was found (absorbance at 414 nm was <0.043 AU). The levels of α-syn in CSF did not differ between the genotypes (data not shown), suggesting that the transgenic expression in oligodendroglia did not lead to significantly enhanced presence of the protein in the CSF. Sequential α-syn extraction of hemibrains revealed overall elevated monomeric α-syn expression in TX-soluble and TX-insoluble fractions derived from PLP-α-syn mice compared to controls, that remained stable throughout the mouse lifespan (Fig. 2b), corroborating the ELISA results. TX-soluble high molecular weight (HMW) oligomeric α-syn species, however, showed an increase in PLP-α-syn mice from 6 months onward. In the TX-insoluble fraction of PLP-α-syn brains, we identified higher levels of monomeric α-syn in all age groups without age-related changes, whereas oligomeric α-syn levels significantly peaked at 12 months of age, but were back to control levels later on at 18 months of age (Fig. 2b).

Analysis of α-syn protein levels in subregions of the MSA mouse brain revealed significantly lower levels of α-syn in the forebrain as compared to midbrain, cerebellum and lower brainstem at all ages (Fig. 2c). Between 2 and 12 months of age, the lower brainstem showed significantly higher levels of α-syn protein expression as compared to all other regions, but this difference to midbrain and cerebellum was lost at 18 months of age (Fig. 2c). The midbrain was the only region that showed a significant age-related increase in the level of α-syn protein expression with aging between 12 and 18 months (*p* < 0.05), while the other regions showed no significant age-related changes (Fig. 2c). We also examined by Western blotting relative α-syn expression in the striatum, SN, hippocampus, lower brainstem and cerebellum in MSA and control mice, at 2 and 12 months of age. MSA mice showed at all ages significantly higher levels of α-syn than the controls, with the highest levels of α-syn expression in lower brainstem, as observed also in the ELISA. However, we found no indication of age-related dynamics in any of the studied regions using this technique (Fig. 2d).

### Progression of motor deficits in PLP-α-syn mice

In order to identify any behavioural phenotypes induced by the oligodendroglial overexpression of α-syn, we used a battery of tests, which revealed progressive motor defects in the PLP-α-syn mice.

**Fig. 3:**
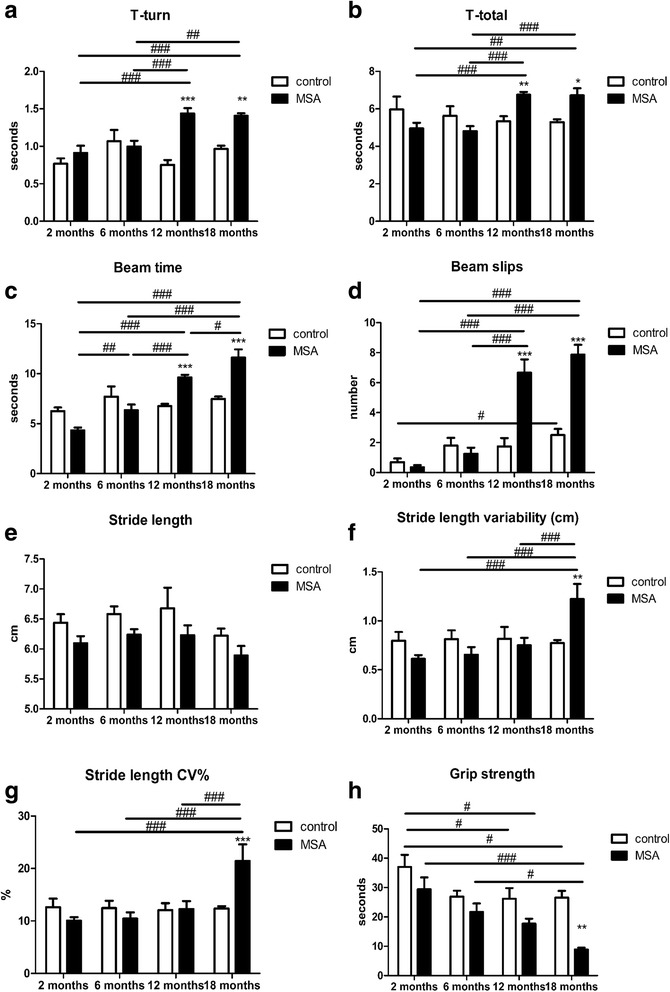
Progressive motor deficits in MSA mice during aging. The performance of the PLP-α-syn and control mice at the pole test is measured by the T-turn time (**a**) and the T-total time (**b**). Two-way ANOVA shows a significant effect of both genotype and aging in pole test (T-turn: effect of genotype F_1,58_ = 24.21, *p* < 0.0001, effect of age F_3,58_ = 6.192, *p* = 0.001, interaction F_3,58_ = 8.093, *p* = 0.0001; T-total: effect of genotype F_1,54_ = 1.097, *p* = 0.2996, effect of age F_3,54_ = 2.895, *p* = 0.0435, interaction F_3,54_ = 6.781, *p* = 0.0002). Post hoc Bonferroni correction shows increase of the T-turn and the T-total time in PLP-α-syn mice at 12 and 18 months of age, with respect to the control mice. Similarly, age- and genotype-related motor function deterioration is observed with the beam test, by measuring the time to go across the beam (**c**) and the number of slips (**d**). Two-way ANOVA shows a significant effect of both genotype and aging in the beam test (crossing time: effect of genotype F_1,65_ = 6.913, *p* = 0.0107, effect of age F_3,65_ = 24.96, *p* < 0.0001, interaction F_3,65_ = 17.89, p < 0.0001; number of slips: effect of genotype F_1,64_ = 37.67, *p* < 0.0001, effect of age F_3,64_ = 33.3, p < 0.0001, interaction F_3,64_ = 17.87, *p* < 0.0001). Post hoc Bonferroni correction shows that the transgenic animals need significantly more time and make significantly more slips than the wild-type controls at 12 and 18 months of age. Gait analysis focused on stride length (**e**) and stride length variability (expressed in cm (**f**) and as a coefficient of variability in percentage (**g**)). A tendency towards shorter stride length is seen in the PLP-α-syn mice compared to the controls (two-way ANOVA with factors genotype and age: effect of genotype F_1,55_ = 9.477, *p* < 0.01, effect of age F_3,55_ = 2.056, *p* > 0.05, interaction F_3,55_ = 0.0517, *p* > 0.05), but sub-group differences are not significant after post-hoc Bonferroni test. Two-way ANOVA shows a significant effect of aging on stride length variability (absolute in cm: effect of genotype F_1,55_ = 0.0254, *p* = 0.8783, effect of age F_3,55_ = 4.304, *p* = 0.0083, interaction F_3,55_ = 5.375, *p* = 0.0025; CV%: effect of genotype F_1,56_ = 1.3, *p* = 0.259, effect of age F_3,56_ = 5.841, *p* = 0.0015, interaction F_3,56_ = 6.161, *p* = 0.0011). Post hoc Bonferroni correction shows that the stride length variability in PLP-α-syn mice is significantly higher than in the controls at 18 months. **h** Grip strength decreases in both control and transgenic animals between 2 and 12 months of age, and in the latter ones this decrement continues till 18 months of age. Two-way ANOVA shows a significant effect of aging and genotype on the hanging time, but no interaction between the factors (effect of genotype F_1,66_ = 20.09, *p* < 0.0001, effect of age F_3,66_ = 8.09, *p* < 0.0001, interaction F_3,66_ = 1.456, *p* = 0.2345). Post hoc Bonferroni correction shows that the grip strength in the MSA mice at 18 months of age is significantly lower than in the wild-type animals. ** *p* < 0.01, ***p < 0.001 versus age-matched controls; # *p* < 0.05, ## p < 0.01, ### *p* < 0.001; for all groups *n* = 7–11

When analysing the effects of the genotype, we found no significant differences between PLP-α-syn and control mice at 2 and 6 months of age with any of the applied behavioural tests (Fig. 3 a-h). Significant deterioration in the motor performance of PLP-α-syn mice as compared to age-matched controls was detected at 12 months of age with the pole test (Fig. 3a,b) as well as with the beam walking test (Fig. 3c, d). At 18 months of age, the PLP-α-syn mice showed worsening of the motor performance as compared to their age-matched controls in all applied tests, including pole test, beam walking, gait analysis and grip strength (Fig. 3 a-d, f-h). Gait analysis indicated a general effect of the genotype on the hindlimb stride length, with PLP-α-syn mice showing shorter strides than control mice (*p* < 0.01). However, post-hoc testing between the individual age groups failed to reach significance (Fig. 3e).

Age-linked motor deterioration in the PLP-α-syn mice was detected between 2 and 6 months of age with the beam test (increased beam crossing time at 6 months, *p* < 0.01, Fig. 3c). At 12 months of age, the PLP-α-syn mice showed significant age-related deterioration in both beam crossing and pole test (Fig. 3a-d). In comparison, up to 12 months of age the control mice showed no aging changes in their motor performance with any of the applied tests (Fig. 3). Finally, at 18 months of age, both control and transgenic mice had significant motor deterioration; however, it was much more prominent in the PLP-α-syn mice. In the control group, age-related change was identified only by the beam walking test (Fig. 3d) and by the analysis of the grip strength (Fig. 3h). In comparison, PLP-α-syn mice at 18 months of age showed aging deterioration in all the applied tests (pole test, Fig. 3a,b; beam walking, Fig. 3c, d; gait analysis, Fig. 3f, g, and grip strength, Fig. 3h). The only parameter that remained stable over time in both control and PLP-α-syn mice appeared to be the stride length (Fig. 3e).

### Progression of neuronal loss in PLP-α-syn mice

**Fig. 4:**
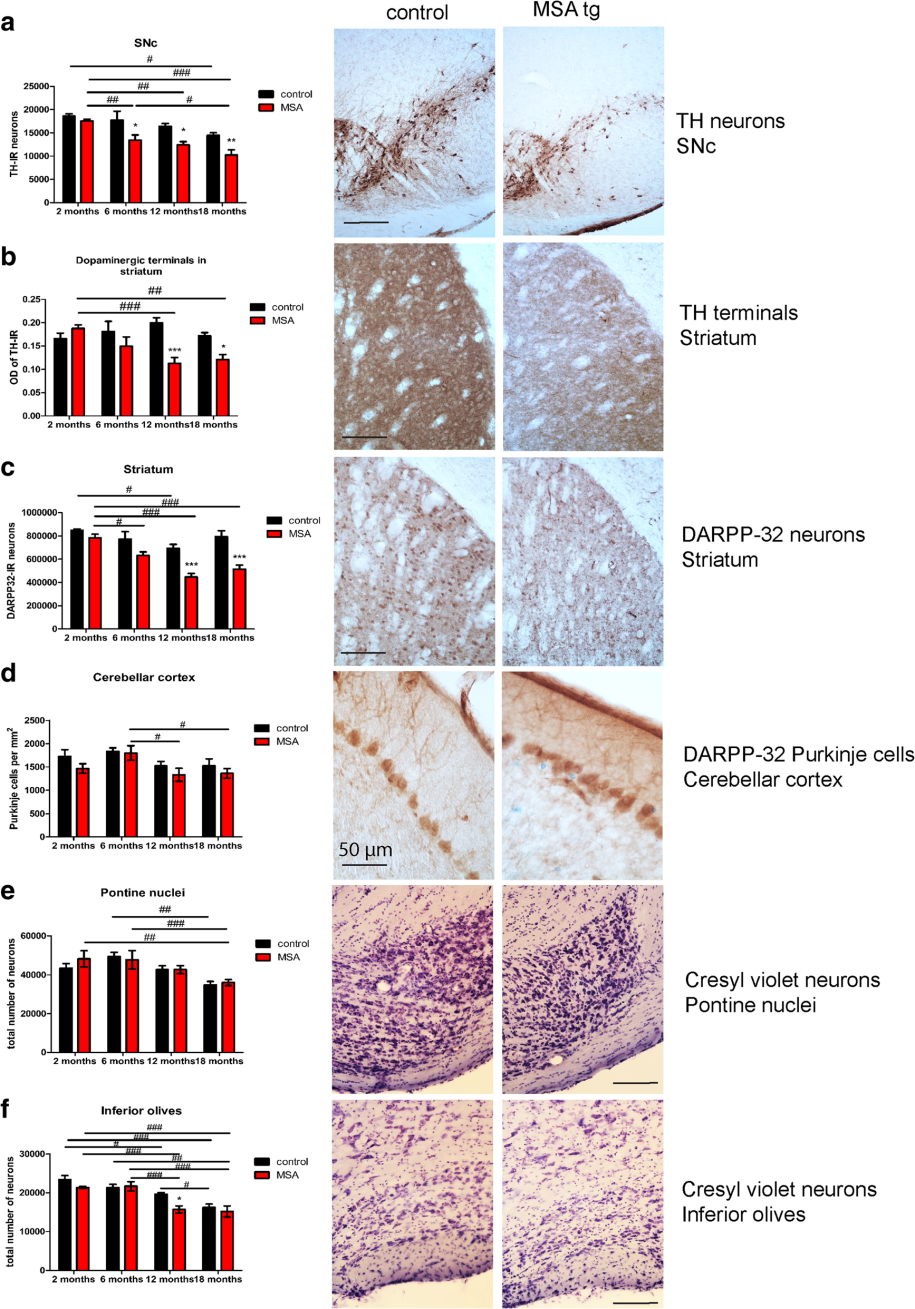
Neuronal loss in MSA mice during aging. **a** In the substantia nigra pars compacta (SNc), two-way ANOVA shows a significant effect of aging and genotype on the neuronal number, but no interaction between the factors (effect of genotype F_1,39_ = 23.58, *p* < 0.0001, effect of age F_3,39_ = 11.37, *p* < 0.0001, interaction F_3,39_ = 1.089, *p* = 0.3653). After post hoc Bonferroni correction, MSA mice present with significant loss of TH neurons with respect to the controls at 6, 12 and 18 months of age. Within the control group, a significant cell loss over time is visible only between 2 and 18 months of age, while for the MSA animals this can be noticed between 2 and 6 months and 6 and 18 months of age. Photomicrographs represent TH-immunoreactivity in SNc at 12 months of age in control and MSA tg mice; (**b**) In the striatum, significant loss of dopaminergic terminals can be seen after post hoc Bonferroni correction at 12 and 18 months of age in MSA mice compared to healthy controls. Progression of dopaminergic terminal loss can be detected between 2 and 12 months and 2 and 18 months of age in MSA mice (two way ANOVA indicates a general effect of genotype but not aging: effect of genotype F_1,40_ = 14.12, *p* = 0.0005, effect of age F_3,40_ = 1.692, *p* = 0.1841, interaction F_3,40_ = 4.825, *p* = 0.0058). Photomicrographs represent TH-immunoreactivity in striatum at 12 months of age in control and MSA tg mice; (**c**) Again post hoc Bonferroni test indicates that, at 12 and 18 months of age, the striatum of the MSA tg mice presents with reduced DARPP-32^+^ medium-spiny neurons compared to the controls, and an age-related decrease of these cells is present as well (two way ANOVA indicates a general effect of both genotype and aging: effect of genotype F_1,40_ = 41.62, *p* < 0.0001, effect of age F_3,40_ = 11.99, *p* < 0.0001, interaction F_3,40_ = 2.925, *p* = 0.0454). Photomicrographs represent DARPP-32-immunoreactivity in striatum at 12 months of age in control and MSA tg mice; (**d**) After post hoc Bonferroni test in the cerebellar cortex, no significant differences in the numbers of Purkinje cells between transgenic and control mice are noticed at any time point, but there is an age-related decrease in these cells’ numbers between 6 and 12 months and 6 and 18 months of age in the transgenic line (two way ANOVA indicates a general effect of aging, but not genotype: effect of genotype F_1,24_ = 3.443, *p* = 0.0758, effect of age F_3,24_ = 4.2, *p* = 0.016, interaction F_3,24_ = 0.2905, *p* = 0.8318). Photomicrographs represent DARPP-32-immunoreactivity of Purkinje cells in the cerebellar cortex at 12 months of age in control and MSA tg mice; (**e**) The pontine nuclei show some age-related decreases in neuronal numbers in both mouse lines, but no differences between the two genotypes at any age (two way ANOVA indicates a general effect of aging, but not genotype: effect of genotype F_1,39_ = 0.3153, *p* = 0.5777, effect of age F_3,39_ = 9.324, *p* < 0.0001, interaction F_3,39_ = 0.4115, *p* = 0.7456). Photomicrographs represent neurons of the pontine nuclei in cresyl violet staining at 12 months of age in control and MSA tg mice; (**f**) In the inferior olives, the only time-point with significant lower neuronal numbers in the MSA than in the control mice after post hoc Bonferroni correction is 12 months of age, possibly indicating accelerated aging in the transgenic inferior olives. There is a clear age-related neuronal loss in both genotypes as indicated (two way ANOVA indicates a general effect of aging and genotype without interaction: effect of genotype F_1,24_ = 6.215, *p* = 0.02, effect of age F_3,24_ = 22.63, *p* < 0.0001, interaction F_3,24_ = 1.867, *p* = 0.1621). Photomicrographs represent neurons of the inferior olives in cresyl violet staining at 12 months of age in control and MSA tg mice. * *p* < 0.05, ** *p* < 0.01, ***p < 0.001 versus control age-matched mice; # *p* < 0.05, ## *p* < 0.01, ###*p* < 0.001. Scale bars, if not otherwise indicated, 200 μm. For all *n* = 4–8

We assessed neuronal loss in brain regions that are typically affected in human MSA and underlie the motor symptoms in this movement disorder, including SNc, striatum, inferior olives (IO), pontine nuclei (PN), and Purkinje cells in the cerebellar cortex. Significant nigral dopaminergic neuronal loss was identified in PLP-α-syn mice at 6, 12 and 18 months of age as compared to age-matched controls (Fig. 4a). An age-related dopaminergic cell loss in PLP-α-syn mice was detected between 2 months and 6 months of age (*p* < 0.01) that further progressed at 18 months of age (*p* < 0.05), but seemed to stabilize between 6 and 12 months of age (*p* > 0.05). In contrast, in control mice a mild age-related cell loss in SNc reached significance only at a very late stage (2 months vs 18 months of age, *p* < 0.05, Fig. 4a). The nigral neuronal loss has been confirmed with cresyl violet staining as in previous observations in the model [18, 58]. Furthermore, we identified reduced density of dopaminergic terminals in the striatum of MSA mice versus controls at the age of 12 and 18 months, as shown by a significant reduction in striatal TH density in PLP-α-syn mice between 2 and 12 or 18 months (*p* < 0.01) (Fig. 4b). By contrast, striatal TH-immunoreactivity did not decline with aging in control mice (Fig. 4b).

Next, we assessed the number of DARPP-32-positive neurons in the striatum and identified significant loss in PLP-α-syn mice as compared to controls at 12 months and 18 months of age (*p* < 0.001, Fig. 4c). While mild age-related loss of DARPP-32-positive striatal neurons was seen in controls (2 vs 12 months, *p* < 0.05), striatal neuronal loss in PLP-α-syn mice occurred earlier (2 vs 6 months of age, *p* < 0.05) and persisted later on (2 vs 12 months of age, *p* < 0.001 and 2 vs 18 months of age, *p* < 0.001).

We identified age-related Purkinje cell loss in PLP-α-syn mice after 6 months of age (6 vs 12 months, *p* < 0.05 and 6 vs 18 months, *p* < 0.05, Fig. 4d). Control mice showed a similar trend without reaching significance. No significant differences between PLP-α-syn mice and controls were detected at any of the studied ages in Purkinje cells’ numbers (Fig. 4d).

We did not observe significant differences in the number of neurons in the PN between PLP-α-syn mice and controls at any of the studied ages (Fig. 4e). We identified similar degree of age-related neuronal loss at the age of 18 months in the PN in both PLP-α-syn mice and controls (6 vs 18 months, respectively *p* < 0.001 and *p* < 0.01).

Finally, in the IO, age-related neuronal loss was evident in both controls (2 vs 12 months, *p* < 0.05, 2 vs 18 months, *p* < 0.001, and 6 vs 18 months, *p* < 0.01) and PLP-α-syn mice (2 vs 12 months, *p* < 0.001, 2 vs 18 months, *p* < 0.001, 6 vs 12 months, *p* < 0.001, and 6 vs 18 months, *p* < 0.001, Fig. 4f). Interestingly, a transient significant difference in the neuronal number in IO between PLP-α-syn mice and controls was detected at 12 months of age (p < 0.05), suggesting an accelerated aging in the presence of oligodendroglial α-syn (Fig. 4f).

### Region-specific progression of microglia activation in PLP-α-syn mice

**Fig. 5:**
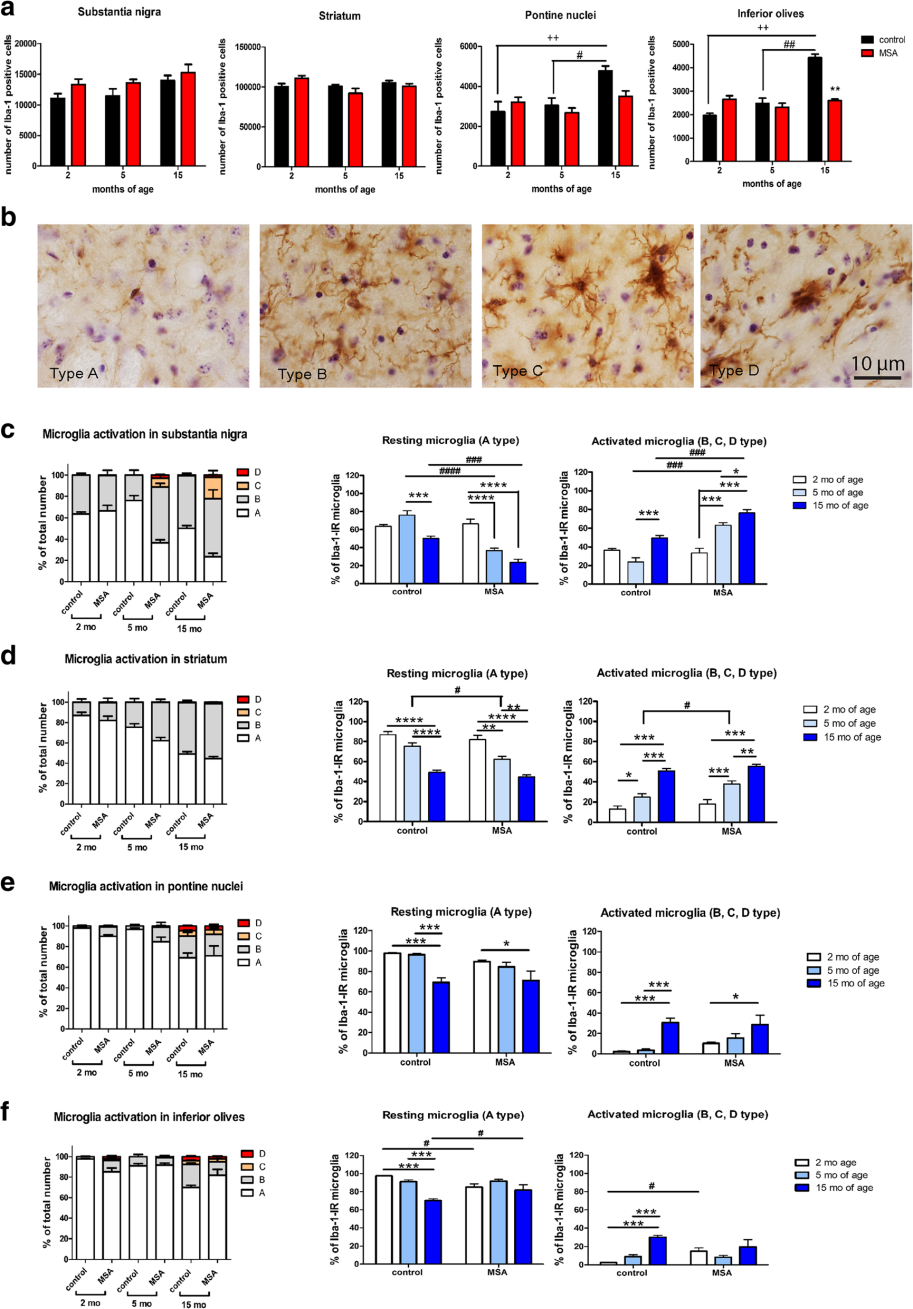
Microglia quantification and profiling in the MSA mice. **a** Sterological analysis of the number Iba-1-positive cells demonstrated no significant differences between control and MSA mice in substantia nigra after Bonferroni correction (two way ANOVA indicates a general effect of age and genotype: effect of genotype F_1,23_ = 6.12, *p* = 0.0211, effect of age F_2,23_ = 3.61, *p* = 0.0432, interaction F_2,23_ = 0.15, *p* = 0.8599). No significant differences between controls and MSA mice were detected after Bonferroni correction in the striatum (two way ANOVA indicates no general effect of age and genotype: effect of genotype F_1,23_ = 0.08, *p* = 0.7812, effect of age F_2,23_ = 3.04, *p* = 0.0671, interaction F_2,23_ = 0.08, *p* = 0.7812). In the pontine nuclei, age-related increase in the number of Iba-1-positive cells was seen in the control, but not in the MSA group after Bonferroni correction (two way ANOVA indicates a general effect of age: effect of genotype F_1,21_ = 1.86, *p* = 0.1866, effect of age F_2,21_ = 8.83, *p* = 0.0017, interaction F_2,21_ = 3.37, *p* = 0.0537). Similar effects were found in the inferior olives; however, at 15 months of age a significant difference between MSA and control mice was found after Bonferroni correction (two way ANOVA indicates a general effect of age and genotype: effect of genotype F_1,22_ = 9.27, *p* = 0.0059, effect of age F_2,21_ = 30.17, *p* < 0.0001, interaction F_2,21_ = 27.05, p < 0.0001) (**b**) Four microglia morphological profiles were distinguished (Iba1 immunohistochemistry and cresyl-violet counterstaining): Type A cells (corresponding to surveillant/homeostatic microglia, and the most abundant phenotype), characterized by a compact and regular shaped nucleus, very thin visible cytoplasm, and long and thin processes without many secondary branches. Type B cells (hyperramified) presented with ramified processes with multiple short branches, and increased amount of cytoplasm around the nucleus as compared to type A. Type C microglia (hyperthrophic) showed even larger cell body with more irregular outline, enlarged nucleus and shorter and thicker processes. Finally, type D cells (amoeboid), which resembled peripheral macrophages, were identified by their amoeboid shape, with a large nucleus and the soma merging with the processes. **c** The stereological counts of each microglia subtype are represented in percentages of the total Iba1^+^ cells counted. In the substantia nigra, the non-homeostatic activated microglia profiles (type B, C and D) were significantly more abundant in the MSA mice than in the control ones at 5 and 15 months of age. Homeostatic microglia (type A) showed a significant reduction from 2 to 5 months and from 2 to 15 months of age in the transgenic animals, while activated microglia were significantly increased from 2 to 15 months of age. In the control mice, a reduction in A cells was seen at 15 months of age (two way ANOVA indicates a general effect of age and genotype: effect of genotype F_1,23_ = 51.42, *p* < 0.0001, effect of age F_2,23_ = 29.42, *p* < 0.0001, interaction F_2,23_ = 19.53, *p* < 0.0001); (**d**) In the striatum, an age-related decrease in A cells, accompanied by an increase in B cells, was observed in both mouse lines. No significant differences were detected between the two at any time point, except for an increase in the percentage of D cells in the MSA mice at 15 months of age (two way ANOVA indicates a general effect of age and genotype without interaction: effect of genotype F_1,23_ = 8.29, *p* = 0.0085, effect of age F_2,23_ = 72.14, *p* < 0.0001, interaction F_2,23_ = 1.1, *p* = 0.35); (**e**) In the pontine nuclei, as well as in the inferior olives (**f**), an increase of activated microglia was visible at 15 months of age, if compared with earlier stages, in both transgenic and control mice (two way ANOVA for the pontine nuclei indicates a general effect of age: effect of genotype F_1,21_ = 2.65, *p* = 0.1184, effect of age F_2,21_ = 3.61, p < 0.0001, interaction F_2,21_ = 1.27, *p* = 0.3008; two way ANOVA for the inferior olives indicates a general effect of age: effect of genotype F_1,21_ = 0.03, *p* = 0.8731, effect of age F_2,21_ = 15.43, *p* < 0.0001, interaction F_2,21_ = 6.11, *p* = 0.0081)

To address changes in microglia population, we performed stereological quantification of Iba1-positive cells. No significant changes with age or genotype were found in the total number of Iba1-positive cells in SN or striatum of PLP-α-syn and control mice after correction for multiple comparisons (Fig. 5a). In the PN we observed a significant increase in the number of Iba1-positive cells in the control mice at 15 months of age as compared to 2 and 5 months of age; this was not seen in the PLP-α-syn mice (Fig. 5a). In the IO there was a significant effect of both age and genotype on the total number of Iba1-positive microglia (Fig. 5a). As observed in the PN, the number of microglia in the IO of 15-months-old control mice increased significantly as compared to 2 or 5 months of age. This was not the case in the IO of PLP-α-syn mice that showed a significantly lower number of microglia at 15 months as compared to controls at the same age (Fig. 5a).

Since microglia may respond to a neurodegenerative process without modifying their number, but instead undergoing a significant change in profile, counting and morphological profiling of Iba1-positive cells were performed simultaneously. To classify the activation profile we adapted a scale previously described for rats and monkeys [3, 48]. Microglia cells were rated to type A, “resting” (homeostatic), B hyper-ramified, C hyper-trophic, or D ameboid, according to the appearance of their processes, nucleus and cell body (Fig. 5b). Thereafter we analysed the percentage of each type in the total Iba1-positive population in each group and area.

In the SN of control mice, the Iba1-positive population was mostly represented by type A and B microglia, with virtually no C or D type microglia detected in this area. We observed no major redistribution in the activation subtypes of microglia with age, except for a mild reduction of Type A (*p* < 0.003) with a shift towards the Type B phenotype at 15 months of age (Fig. 5c). In contrast, in SN of PLP-α-syn mice, the presence of the type C and D became apparent at 5 months of age and the percentage of the activated subtypes (B, C and D) showed a significant increase (p < 0.003 at 15 vs 2 months of age) in parallel to the significant reduction of homeostatic microglia (type A) at the age of 5 and 15 months (for both p < 0.003 as compared to 2 months of age; Fig. 5c). Respectively, there was a significant increase of the activated microglia subtypes (B, C and D) in SN of PLP-α-syn as compared to control mice at 5 and 15 months of age (Additional file 1: Table S1 and Table S3).

**Fig. 6:**
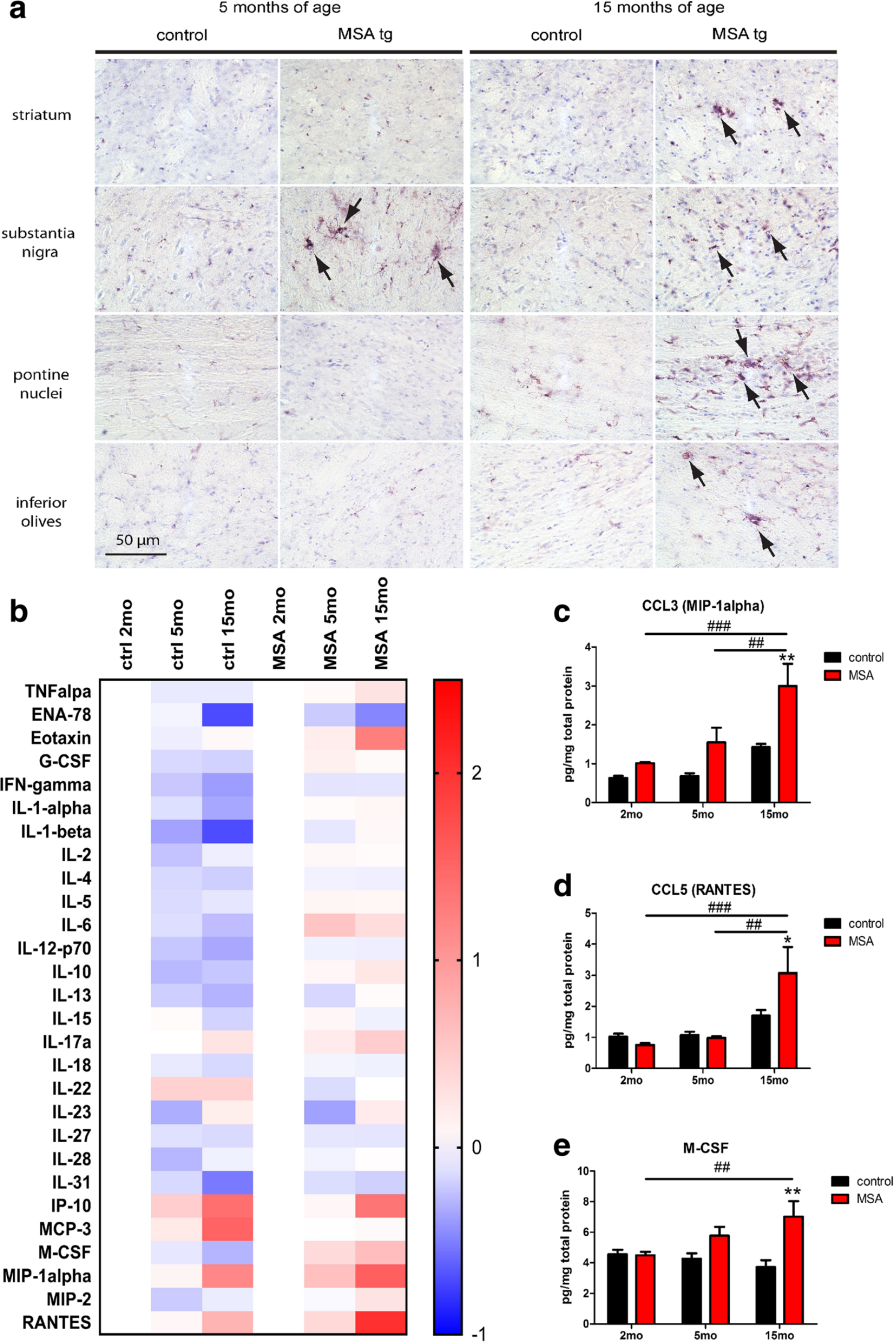
CD68 positive microglia and cytokine/chemokine levels in the in MSA mouse brain. **a** CD68 immunohistochemistry reveals the presence of abundant CD68^+^ activated microglia cells with C type or D type morphology in 5 months old MSA substantia nigra (arrows). At 15 months of age, robust microglial activation with CD68^+^ profiles (arrows) is detected in all regions of the MSA mouse brain and not so prominently in healthy controls. **b** Heat map comparing the log2 fold change in cytokine and chemokine expression in the brains of control and MSA mice at 5 and 15 months of age, as referenced to mice of the same genotype at 2 months of age, demonstrates the changes in the general neuroinflammatory profile with aging in the two genotypes. **c**, **d**, **e** As measured by an immunoassay, the protein levels of CCL3, CCL5 and M-CSF show an increase over time in the MSA mice, with significant higher levels than in the control animals at 15 months of age. (two-way ANOVA for CCL3 with factors genotype and age: effect of genotype F_1,18_ = 16.57, *p* = 0.0007, effect of age F_2,18_ = 13.36, *p* = 0.0003, interaction F_2,18_ = 2.24, *p* = 0.1352; two-way ANOVA for CCL5 with factors age: effect of genotype F_1,18_ = 1.35, *p* = 0.2611, effect of age F_2,18_ = 10.81, *p* = 0.0008, interaction F_2,18_ = 3.22, *p* = 0.064; two-way ANOVA for M-CSF with factors genotype: effect of genotype F_1,18_ = 12.12, *p* = 0.0027, effect of age F_2,18_ = 1.15, *p* = 0.3382, interaction F_2,18_ = 4.56, *p* = 0.0249)

We identified comparable percentage distribution of homeostatic and activated microglia in the striata of age-matched PLP-α-syn and control mice (Fig. 5d). Type A microglia in striatum showed significant age-related decrease in both PLP-α-syn and control mice (*p* < 0.003 in 2- vs 15-months-old); however, accelerated reduction in the percentage of homeostatic microglia was observed at 5 months of age in PLP-α-syn mice (2- vs 5-months-old, *p* < 0.003) as compared to age-matched controls (Fig. 6d). Similarly, the increase of B type microglia was significant with age (2- vs 15-months-old, p < 0.003) and comparable in the striata of PLP-α-syn and control mice (Fig. 5d). However, the increase in the B type microglia was detectable earlier in PLP-α-syn mice (2- vs 5-months, *p* < 0.003). C and D type activated microglia each comprised about 1% of the total microglia population in the striatum of PLP-α-syn mice at 15 months of age and were practically absent in age-matched controls (Fig. 5d). Microglia activation profiles in striatum between control and PLP-α-syn mice were only significantly different at 15 months of age, linked to the presence of D type microglia activation in PLP-α-syn striatum (Additional file 1: Table S1 and Table S3).

In PN there was a clear shift from homeostatic (type A) to activated (Type B, C, D) microglia at the age of 15 months (Fig. 5e), with no significant differences between control and MSA mice (Additional file 1: Table S1 and Table S3). A similar picture was identified in the IO. A clear shift towards activated phenotypes of microglia was seen at 15 months of age, which was more prominent in control than in PLP-α-syn mice (Fig. 5f).

In order to characterize the phenotypic response of the microglia, we analysed the expression of MHCII and CD68 by immunohistochemistry in the selected brain regions. While Iba-1 is constitutively expressed in all microglia, MHCII and CD68 expression is upregulated during α-syn induced neurodegeneration [47]. MHCII is related to antigen processing, and it therefore confers antigen presenting cell identity to the microglia. Our analysis showed that only very few, single MHCII-positive cells are present in the brains of 5- and 15-months-old PLP-α-syn mice (data not shown). On the other hand, CD68 expression is considered an indicator of phagocytic activity of microglia [68, 76]. This microglial lysosomal protein showed typical distribution in an intracytoplasmic dot-like pattern, consistent with the labelling of lysosomes within microglia (Fig. 6a). Furthermore, CD68 immunostaining revealed increased levels of microglial phagocytic activity in the SN of PLP-α-syn mice at 5 months of age, which further extended in all the studied regions at 15 months of age in the PLP-α-syn brain. In contrast, in healthy age-matched mice, only few or none CD68-positive cells were identified (Fig. 6a).

To further characterize the immune response in the PLP-α-syn mice, we used a multi-panel to analyse levels of 36 cytokines and chemokines in whole brain extracts. A heatmap depicting the overall age-related changes of cytokines/chemokines in control and PLP-α-syn mice showed different aging profiles between the genotypes (Fig. 6b). The analysis revealed age-related increase in the levels of CCL3 (MIP-1alpha), CCL5 (RANTES) and the macrophage colony stimulating factor (M-CSF) in PLP-α-syn mouse brains, reaching significant difference from age-matched controls at 15 months of age (Fig. 6c-e). The brain concentration of the remaining analytes showed no significant effect of either genotype or age (Additional file 1: Table S2).

Since the neuroinflammatory response may be related to astrogliosis in the advanced stages of the disease we wanted to address this feature in the neuropathology of the PLP-α-syn mouse too. We found age-related increase of GFAP OD in both control and transgenic mice, however no significant increase of astroglial activation was evident in the presence of oligodendroglial α-syn pathology up to 18 months of age in the MSA mice (Additional file 1: Figure S1).

### Region-specific changes of neurotrophic support in PLP-α-syn mice

**Fig. 7:**
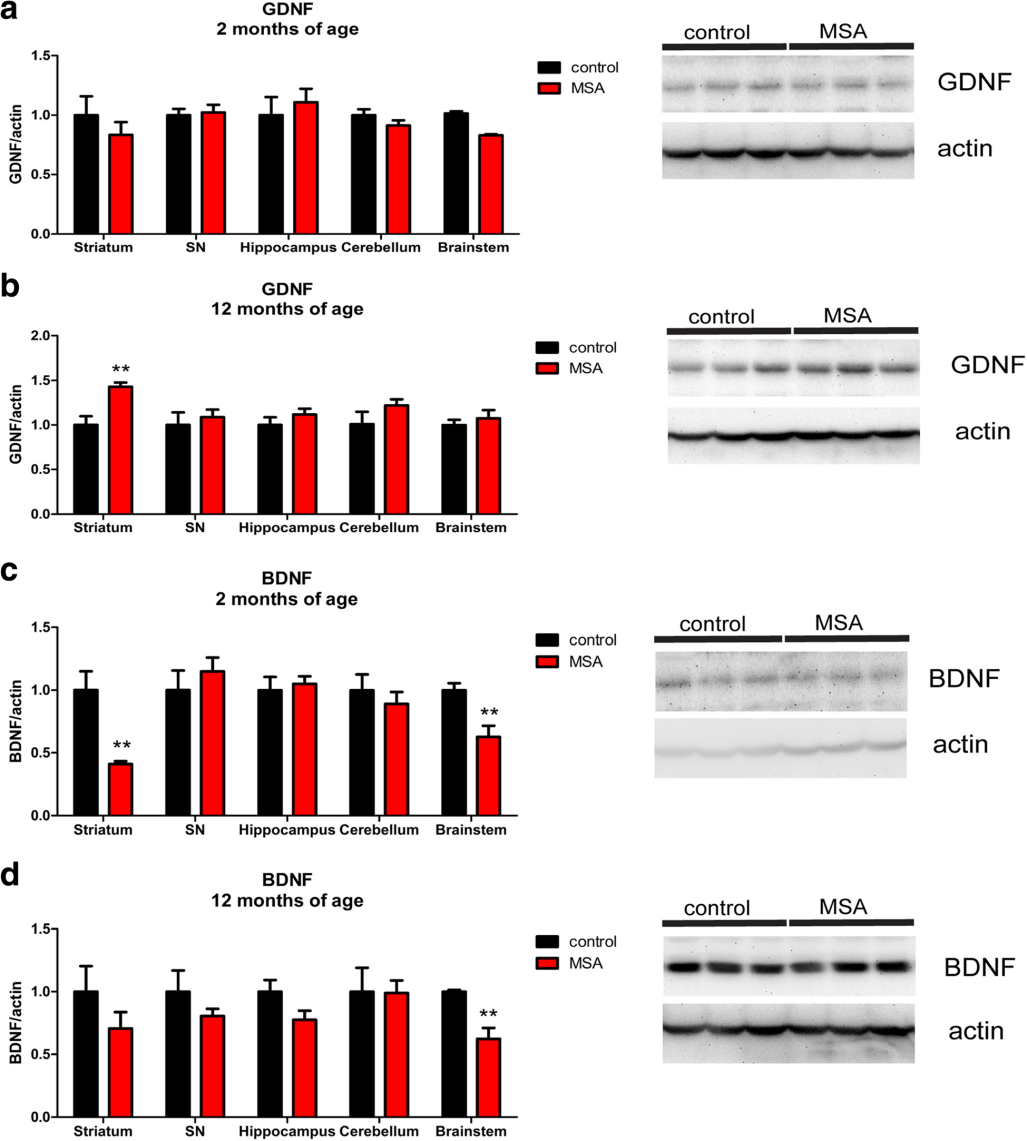
Neurotrophic support in PLP-α-syn mice. **a** GDNF protein levels of 2 months old MSA mice as compared to the controls by two way ANOVA indicated no general effect of region and genotype: effect of genotype F_1,50_ = 1.09, *p* = 0.30224, effect of region F_4,50_ = 0.83, *p* = 0.5118, interaction F_4,50_ = 0.93, *p* = 0.4564), (**b**) At 12 months of age, GDNF levels get higher in the striatum of the transgenic mice than in the controls, possibly reflecting compensatory glial activation at this stage (two way ANOVA indicates a general effect of the genotype: effect of genotype F_1,49_ = 9.58, *p* = 0.0.0033, effect of region F_4,49_ = 1.27, *p* = 0.2964, interaction F_4,49_ = 1.25, *p* = 0.3032). **c** BDNF shows decreased protein levels in the striatum and brainstem of MSA mice compared to age-matched counterparts at 2 months of age (two way ANOVA indicates a general effect of region and genotype: effect of genotype F_1,50_ = 6.88, *p* = 0.0115, effect of region F_4,50_ = 4.2, *p* = 0.0052, interaction F_4,50_ = 4.2, *p* = 0.0052); (**d**) At 12 months of age, the difference in the brainstem remains, while the one in the striatum loses its significance, possibly linked to compensatory mechanisms related to glial activation (two way ANOVA indicates a general effect of genotype: effect of genotype F_1,49_ = 7.42, *p* = 0.0089, effect of region F_4,49_ = 0.58, *p* = 0.6771, interaction F_4,49_ = 0.58, *p* = 0.6771); ** *p* < 0.01, ***p < 0.001 compared to age-matched healthy controls. Band size: BDNF, 14 kDa; GDNF, 15 kDa; actin, 40 kDa

To detect whether oligodendroglial overexpression of α-syn under the control of the PLP promoter in PLP-α-syn mice had an effect on the production of neurotrophic factors, as suggested by findings in a different transgenic mouse model of MSA, the MBP-α-syn mouse [65], we measured the levels of protein expression of BDNF and GDNF. The analysis was performed in several brain areas of young non-symptomatic animals at 2 months of age and aged symptomatic MSA mice at 12 months of age, and compared to healthy controls of the same age by multivariate t-test (Fig. 7). At 2 months of age, a significant decrease of GDNF levels was identified in the lower brainstem of MSA mice (*p* < 0.001), but not in the other studied regions (Fig. 7a). At the same age, BDNF levels were significantly reduced in MSA striatum (*p* = 0.003) and lower brainstem (*p* = 0.005) as compared to control levels (Fig. 7c). At 12 months of age, GDNF levels were increased in the MSA striatum (*p* = 0.004) as compared to controls (Fig. 7b). Simultaneously, lower BDNF levels were maintained in MSA lower brainstem during aging (*p* = 0.003 as compared to controls, Fig. 7d).

### No evidence of myelin dysfunction in symptomatic PLP-α-syn mice

As robust indications of neurodegeneration and motor dysfunction were detected in PLP-α-syn mice at the age of 12 months, we performed histological and biochemical analysis of myelination at this age. No demyelinated lesions were seen with classical luxol fast blue staining (data not shown). There was no significant difference in MBP, MOG and p25α protein expression levels between hemibrains of PLP-α-syn mice and healthy controls as measured by western blotting (Additional file 1: Figure S2), suggesting that the overexpression of α-syn in oligodendrocytes under the PLP promoter did not have any significant early impact on the production of myelin proteins in this model, and could hardly be causative for the early neurodegeneration occurring in the PLP-α-syn brain long before the age of 12 months.

## Discussion

### Relevance of the PLP-α-syn mouse phenotype to human MSA clinical presentation and neuropathology: Insights into disease progression

Previous studies in the PLP-α-syn transgenic mouse model of MSA have identified prominent GCI-like pathology [32], loss of dopaminergic neurons in SNc [18, 54, 57, 58], early microglial activation [58], and motor deficits [18, 57]. Furthermore, MSA-like non-motor features including neurogenic bladder dysfunction [7], cardiovascular autonomic failure [35, 61], breathing variability [19], and REM sleep behaviour disorder [26] have been reported in the PLP-α-syn mouse in relation to neurodegeneration affecting selected brainstem nuclei including the laterodorsal tegmental nucleus, pedunculopontine tegmental nucleus, nucleus ambiguus, the pontine micturition centre, raphe nuclei, as well as centres in the spinal cord including the parasympathetic outflow in the intermediolateral columns and the Onuf’s nucleus. The objective of the current study was to characterize the motor phenotype and its underlying neuropathology in aging PLP-α-syn mice. Here we report progression of motor deficits with first mild signs at 6 months of age and further deterioration and robust detection of decline versus control mice at 12 and 18 months of age. Therefore, the functional phenotype of the PLP-α-syn transgenic mouse replicates the natural history of MSA with early non-motor symptoms like neurogenic bladder dysfunction and REM sleep behaviour disorder followed by a progressive motor syndrome [13]. The PLP-α-syn mouse model gave us the unique opportunity to follow the onset and progression of neuropathological events that underlie the MSA-like phenotype with aging.

**Fig. 8:**
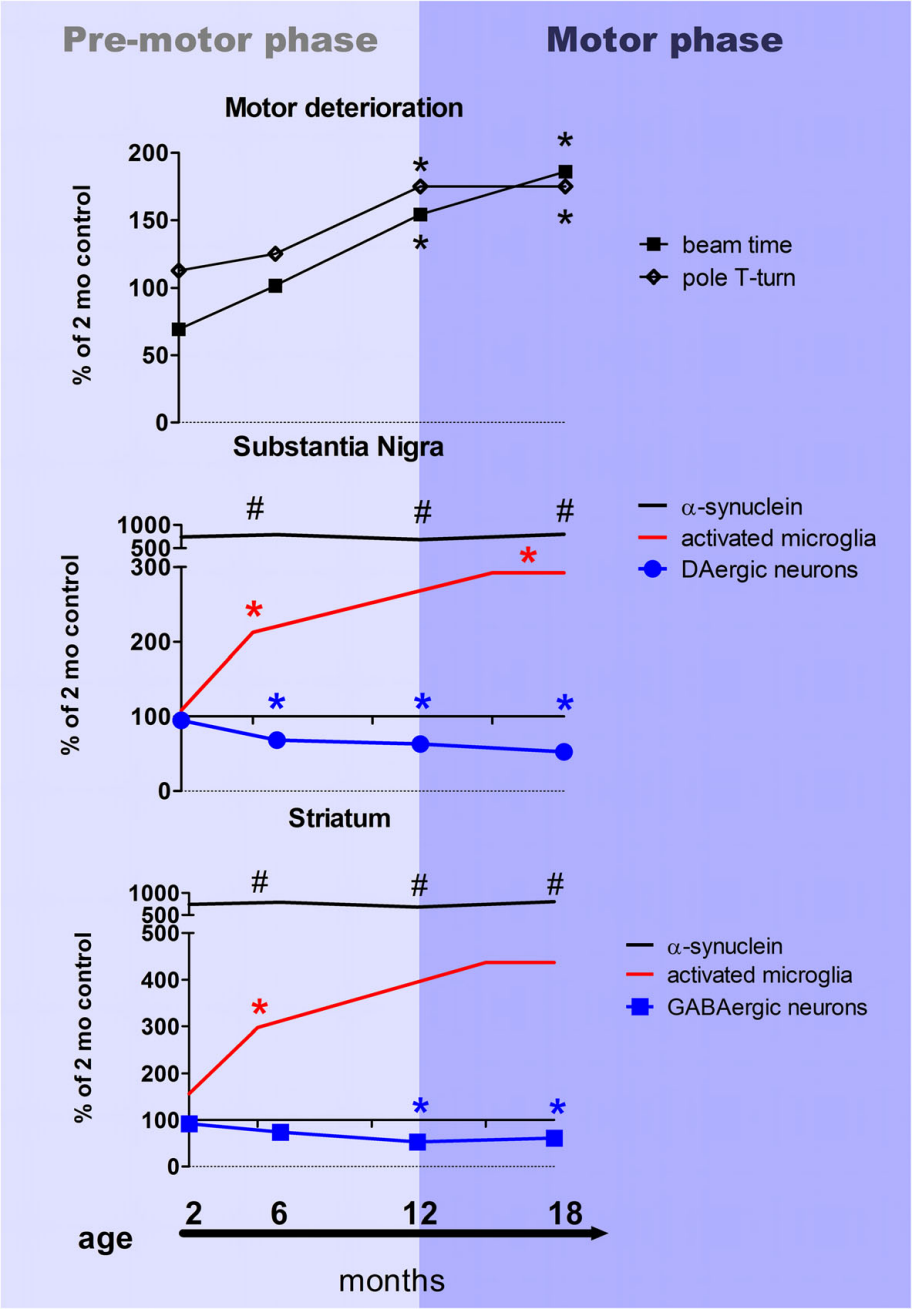
The progression of the motor phenotype and the underlying pathogenic events in the PLP-α-syn transgenic mouse. A robust motor phenotype in the PLP-α-syn (upper panel) is measured at 12 months when the symptomatic motor phase starts. As evident from the brain analysis the neurodegenerative process starts much earlier than the symptomatic presentation of the motor syndrome. The total level of α-syn in the brain of the PLP-α-syn mice remains stable over time, however at 6 months of age soluble oligomeric α-syn species are formed (#). Approximately at the same time strong microglial activation is detected in the substantia nigra (middle panel) that is followed by loss of dopaminergic neurons. The dopaminergic degeneration progresses further and leads to loss of dopaminergic terminals in the striatum as well as loss of striatal GABAergic medium spiny neurons (lower panel) at the time of onset of the motor symptoms. Data are presented as percentage of control wild-type 2 months old mice. Asterisk (*) indicates the time points when significant differences between age-matched wild-type and PLP-α-syn mice occur as presented in detail in the study

The PLP-α-syn mouse, like other existing MSA models [4, 36, 49, 75], is based on the strategy of targeted overexpression of human α-syn in oligodendrocytes, which corresponds to the hypothesis raised by Asi and co-workers that MSA oligodendroglia may express more SNCA mRNA than control oligodendrocytes [2]. The accumulation of α-syn in oligodendrocytes is the trigger of the observed phenotype and all the pathogenic events that follow in the PLP-α-syn mouse. Interestingly, the total amount of α-syn in the brain remains stable over time and cannot account for the disease progression in the PLP-α-syn mouse. However, the occurrence of soluble α-syn oligomers in the brain seems to coincide with the time when microglial activation is first detected in the substantia nigra, suggesting specific hyper-reactivity of the nigral microglia to α-syn oligomers. Acting together, these factors seem to trigger early dopaminergic neuronal loss in SNc. This notion is corroborated by earlier observations suggesting that suppression of microglial activation with minocycline between 2 and 4 months of age can rescue nigral neurons in the PLP-α-syn brain [58]. The resulting 25–30% dopaminergic nigral loss between 2 and 6 months of age may account for the mild deterioration in the beam test performance of the PLP-α-syn mice in this timespan. However, the time of robust motor deterioration (measurable with both the beam and pole test) overlaps with the time of the occurrence of striatal neuronal loss and loss of striatal dopaminergic terminals in the PLP-α-syn mouse (Fig. 8) – a finding that corroborates a previous report of reduction of striatal dopamine turnover in aged PLP-α-syn mice [18]. Therefore, the clinically overt motor phase starts at 12 months of age in the PLP-α-syn model, which provides a correlate to onset of motor symptoms and time of first diagnosis in MSA patients [23]. As demonstrated here, the motor phenotype in the PLP-α-syn transgenic mouse reflects progressive SND with relative sparing of the olivopontocerebellar motor circuits. Considering the additional autonomic brainstem involvement, the PLP-α-syn mouse therefore replicates the MSA-P subtype [13].

### Insights into the mechanisms of region-specific MSA-P-like neurodegeneration

In search of the mechanisms that may underlie selective SND in the PLP-α-syn mouse, we addressed several possible candidates, including α-syn protein species and levels, oligodendroglial dysfunction, trophic support and neuroinflammation.

#### Oligomeric α-syn and selective microglial activation as triggers of SND

We identified, by both histological and biochemical analyses, region-specific differences in the level of human α-syn expression in the PLP-α-syn brain, with highest protein levels in the brainstem. These region-specific differences can be explained with the typical caudal-to-rostral spatial distribution of PLP, with highest expression in the adult brain in the hindbrain, midbrain and the white matter tracts [72]. However, the region-specific levels of α-syn in the PLP-α-syn transgenic mouse cannot account for the selective neuronal loss observed in the striatonigral region, but the relative sparing of the olivopontocerebellar pathways. Age-related changes in the solubility and oligomerization of α-syn were further detected in the brains of PLP-α-syn mice. For the first time we report here an increase in the TX-soluble oligomeric α-syn species between 2 and 6 months that remains stable over the further lifespan of the animals. Interestingly, the time of oligomeric α-syn increase and dopaminergic nigral loss coincides. Therefore, the greater toxicity of oligomeric forms may contribute to the initiation rather than the progression of the neurodegenerative process in the PLP-α-syn mouse, as previously suggested in PD models [44, 73]. Despite the cell-to-cell transfer, only oligodendroglial aggregates, but no actual accumulation of α-syn in neurons was identified in the PLP-α-syn brain, suggesting that the MSA-P-like selective neuronal loss is not dependent on specific intra-neuronal accumulation of α-syn, and other mechanisms might be responsible for the progressive disease phenotype.

The PLP-α-syn mouse presents with significant microglial activation that accompanies the α-syn accumulation in oligodendrocytes [57, 58], similar to human MSA [29, 45]. The heterogeneous nature of microglia within the brain has been recently shown using single cell and population genetic analysis [12, 38]. Our current results demonstrate that SN microglia shows the strongest and earliest response to the α-syn overexpression in the PLP-α-syn mouse. Both Iba1 morphological profiling and CD68 immunohistochemistry point towards SN-specific responses of microglia that may mediate early neuronal loss in the region. Indeed, the selective vulnerability of SN dopaminergic cells to inflammatory events has been long proposed [25, 62] and corroborated in this study. The causative role of the α-syn-triggered microglial activation for the nigral neuronal loss in the PLP-α-syn mouse is supported by previous data demonstrating that early suppression of microglial activation between 2 and 4 months of age can rescue nigral dopaminergic neurons in these mice [58]. Furthermore, we observed increased levels in M1-associated chemokines: CCL3/MIP-1a and CCL5/RANTES and the latter has been recently shown to have a key role in dopaminergic toxicity [9]. However, we also found increased M-CSF, a cytokine associated to M2 signalling in macrophages [37], which suggests a complex ongoing inflammatory process and disrupted balance of immune signals being essential for neuronal survival. These data seem to further support the dual role of microglial activation which we and others have previously discussed [15, 16, 54, 66]. The fine balance between the beneficial (clearance of α-syn) and detrimental effects (pro-inflammatory toxic signalling) of microglial activation may interfere with the degenerative process and may present an important target to selectively modulate disease progression in MSA and other synucleinopathies. On a side note, astrogliosis was not found to be a major contributor to the genotype-specific neuroinflammatory profile of the PLP-α-syn mouse (Additional file 1: Figure S1) suggesting that astrogliosis may only represent a late event of the disease progression, but not a strong contributor of early disease progression.

We also observed progressive age-related changes of microglia, irrespective of the genetic background of the animal indicative of microglia senescence [27]. However, in the PN and IO of PLP-α-syn mice, the additional ongoing disease process seems to interfere with the proliferative activity and possibly the normal function of microglia. The increased exposure to α-syn oligomers and/or the decrease of GDNF and BDNF in the area may contribute to this specific change in the senescent chronically activated microglia of PLP-α-syn mice. Intriguingly, a recent study showed phagocytic defects of macrophages towards oligomeric α-syn in parallel to stronger pro-inflammatory response related to aging [6]. The relevance of disrupted senescence of microglia has not been addressed to date in human α-synucleinopathies, whereas all experimental data summarized here point towards the importance of this pathogenic factor. Furthermore, knowledge on the changes in microglia senescence in α-synucleinopathies may give an explanation of the failure of clinical trials which target microglial activation in these disorders [11, 39, 40].

#### Disrupted trophic support or dysmyelination cannot explain SND in the PLP-α-syn mouse

Ubhi et al. suggested decreased neurotrophic factors as a candidate mechanism of neurodegeneration in MSA [65]. In the PLP-α-syn mouse we observed significant reductions of BDNF in the lower brainstem (including pons and medulla oblongata - the region with highest α-syn protein expression in this model), but not in the midbrain and the degenerating SN of these mice. Therefore, neurotrophic changes do not seem to be accountable for the early nigral dopaminergic loss in this model. It is plausible that the observed early neurotrophic deficits in the lower brainstem (pons and medulla) of PLP-α-syn mice are responsible for the neuronal loss in central autonomic nuclei linked to the non-motor phenotype, including loss of cholinergic neurons in the nucleus ambiguus, the laterodorsal tegmental nucleus, and the pedunculopontine nucleus, and glutamatergic neurons of the Barrington’s nucleus (the pontine micturition centre) [7, 35, 61]. However, the trophic factor deficit in the lower brainstem of the PLP-α-syn mice does not trigger neuronal loss in the PN (griseum pontis) projecting predominantly to the cerebellum and involved in the control of motor activity [50].

Finally, demyelination is not typically found in the PLP-α-syn model featuring SND in contrast to the MBP-α-syn mouse model characterized by neuronal loss in the neocortex [49]. Although principally based on a similar approach –the targeted overexpression of human full-length α-syn in oligodendrocytes-, the two MSA models differ in phenotype and selectivity of the neurodegenerative process. The PLP-α-syn mouse represents a model of typical MSA-P with SND and neuronal loss in non-motor brainstem centres of cardiovascular, micturition, respiration and sleep control, linked to changed microglial responses in the basal ganglia and deficient neurotrophic support in the lower brainstem. In contrast, the MBP-α-syn mouse presents with axonal degeneration in the basal ganglia, brainstem and cerebellum linked to widespread demyelination and neuronal loss in the cortex, associated with neurotrophic deficits. In summary, the two models represent the heterogeneous pathology of MSA and suggest that dependent on the α-syn-triggered secondary pathology (microglial activation, demyelination), variable disease phenotypes may arise.

## Conclusions

**Fig. 9:**
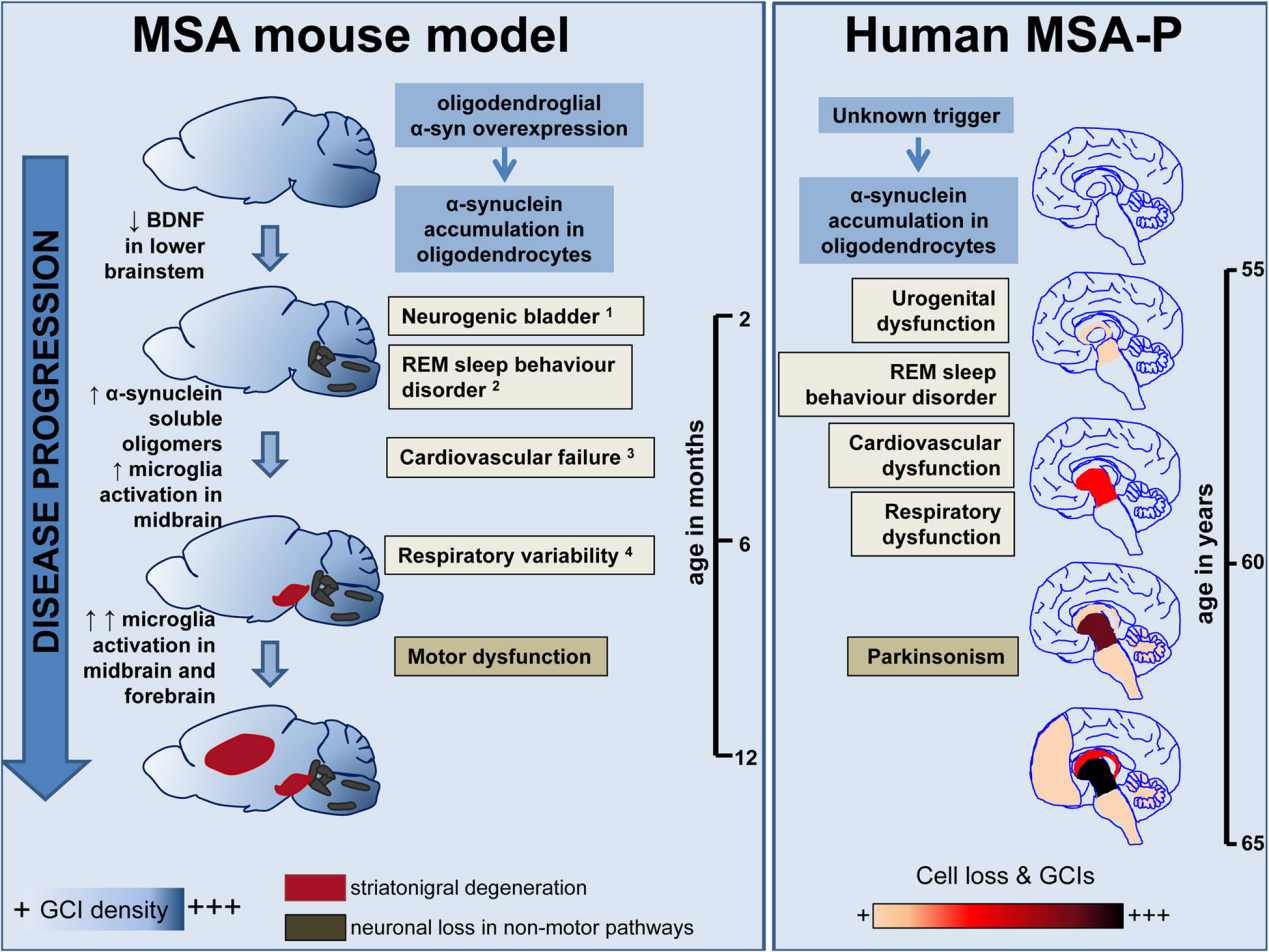
The natural history of MSA-P in PLP-α-syn mice and humans in parallel. In human Parkinson variant MSA, the aetiology and early pathogenic events, presumably asymptomatic, remain unknown. The clinical diagnosis is usually determined with the onset of the motor symptoms (parkinsonism) and at that stage, retrospectively one identifies the pre-history of non-motor dysfunction including urogenital dysfunction, cardiovascular dysfunction, REM sleep behaviour disorder, and respiratory dysfunction [13]. Based on neuropathological studies, Halliday and co-workers proposed progression of the pathology in MSA-P including neuronal loss and GCIs in the basal ganglia and later on spreading in other brain areas [24]. Definite diagnosis of MSA and insights into the clinicopathological correlations is only possible post-mortem, however the identified spreading of GCIs, neuronal loss and gliosis at this end-stage of the disease can hardly answer the question how the disease progresses and what are the underlying mechanisms. Here we describe the disease progression in MSA mice (the PLP-α-syn transgenic mouse) that are genetically engineered to present oligodendroglial human α-syn overexpression leading to GCI-like pathology in the brains even at very early age. The GCI-load does not change with aging but differs among different brain areas (increasing from rostral to caudal regions, as shown by the colour gradient, see Fig. 2). MSA mice show a very early decrease in BDNF levels in the lower brainstem (see Fig. 7), which may be linked to the observed early degeneration of the brainstem nuclei involved in the regulation of autonomic functions, as indicated by the onset of neurogenic bladder dysfunction (^1^ [7]), REM sleep behaviour (^2^ [26]), cardiovascular failure (^3^ [35] and respiratory variability (^4^ [19]). Later on during the disease progression in the MSA mouse, the occurrence of soluble α-syn oligomeric species in the brain is accompanied by strong microglia activation which can be observed specifically in the midbrain (See Figs. 5 and 6), and leads to neuronal loss of dopaminergic neurons in SNc (see Fig. 4) [58]. Further on, the neurodegeneration spreads and involves other regions, including the striatum (Fig. 4). At this time robust progressive motor dysfunction (Fig. 3), resulting from the SND can be identified in the MSA mice. Notably, olivopontocerebellar atrophy, which underlies the cerebellar features in human MSA, can be triggered in the PLP-α-syn transgenic mouse only after exposure to exogenous stress like mitochondrial or proteasomal dysfunction [56, 57], but is not apparent due to the α-synuclein overexpression in oligodendrocytes per se. The striking overlap between the behavioural phenotype of the MSA mouse model and the clinical presentation in MSA-P patients suggests that the PLP-α-Syn transgenic mouse is a valuable preclinical tool to get insights into the pathogenesis of the disease and identify and validate targets of disease modification

The PLP-α-syn mouse model has been widely used in the past 10 years as a template for translational research on MSA. PLP-α-syn mice replicate a number of MSA features, including motor impairment, glial α-syn pathology and MSA-like neurodegeneration. However, the effects of aging have only been incompletely addressed. Our study of the PLP-α-syn model shows progressive SND, the key neuropathological substrate of MSA-P [14]. Due to its relevance to the human disease (Fig. 9), the model provides the unique opportunity to study longitudinally the pathogenic events during the disease progression of MSA. Our data suggest that neuroinflammation triggered by targeted oligodendroglial α-syn overexpression is a leading pathogenic factor for nigral degeneration. Further studies will be needed to address the possible role of disrupted neurotrophic support in lower brainstem and its association with brainstem pathology accounting for MSA-like non-motor features [7, 26, 35, 61]. In summary, the PLP-α-syn mouse is a model that replicates progressive MSA-P [13] and provides a useful preclinical tool for tailored stage-dependent therapeutic screening.

## Additional file

Additional file 1: Figure S1. Progression of astrogliosis in control and PLP-α-syn mice was assessed by meaning GFAP OD. Although earlier initiation of astrogliosis, between 2 and 6 months of age, was seen in the transgenic mice, there was no significant difference between the age-matched MSA and control animals. However, a numeric increase of astrogliosis can be seen in the PLP-α-syn mice, suggesting that further analysis of astroglial activation may be warranted in the model in relevance to the postmortem finding in human MSA. **Figure S2.** Western blot analysis does not show any major differences between control and MSA mice as of MBP, MOG and p25α protein levels at 12 months of age when the major phenotype is detectable (unpaired t-test for MBP: t=1.997, df=8, *p*=0.0809; unpaired t-test for MOG: t=1.252, df=8, *p*=0.2458: unpaired t-test for p25α: t=0.3305, df=8, p=0.7495). **Table S1.** The table represents the changes in the different subtypes of microglia (A, B, C and D) in the different brain regions. Legend: ↑↑ significant increase in MSA (*p*<0.016); ↓↓ significant decrease in MSA (*p*<0.016); ns, no significant difference. Statistical analysis was done by repeated measures ANOVA with post hoc Bonferroni test with correction for multiple comparisons. **Table S2.** The table represents the concentrations of the different cytokines and chemokines measured in the brains of control and MSA mice at 2, 5 and 15 months of age by an immunoassay. Data are presented as mean±SD. **Table S3.** Numbers of microglia subtypes in different brain regions. SN, substantia nigra; PN, pontine nuclei; IO, inferior olives. (PDF 3788 kb)
