# Supplementary material for: Progressive striatonigral degeneration in a transgenic mouse model of multiple system atrophy: translational implications for interventional therapies
Source: Acta Neuropathol Commun. 2018 Jan 3;6:2. doi: 10.1186/s40478-017-0504-y (PMC5753576; doi:10.1186/s40478-017-0504-y)
Supplement: Additional file 1: Figure S1. — Progression of astrogliosis in control and PLP-α-syn mice was assessed by meaning GFAP OD. Although earlier initiation of astrogliosis, between 2 and 6 months of age, was seen in the transgenic mice, there was no significant difference between the age-matched MSA and control animals. However, a numeric increase of astrogliosis can be seen in the PLP-α-syn mice, suggesting that further analysis of astroglial activation may be warranted in the model in relevance to the postmortem finding in human MSA. Figure S2. Western blot analysis does not show any major differences between control and MSA mice as of MBP, MOG and p25α protein levels at 12 months of age when the major phenotype is detectable (unpaired t-test for MBP: t=1.997, df=8, p=0.0809; unpaired t-test for MOG: t=1.252, df=8, p=0.2458: unpaired t-test for p25α: t=0.3305, df=8, p=0.7495). Table S1. The table represents the changes in the different subtypes of microglia (A, B, C and D) in the different brain regions. Legend: ↑↑ significant increase in MSA (p<0.016); ↓↓ significant decrease in MSA (p<0.016); ns, no significant difference. Statistical analysis was done by repeated measures ANOVA with post hoc Bonferroni test with correction for multiple comparisons. Table S2. The table represents the concentrations of the different cytokines and chemokines measured in the brains of control and MSA mice at 2, 5 and 15 months of age by an immunoassay. Data are presented as mean±SD. Table S3. Numbers of microglia subtypes in different brain regions. SN, substantia nigra; PN, pontine nuclei; IO, inferior olives. (PDF 3788 kb) [file 40478_2017_504_MOESM1_ESM.pdf]

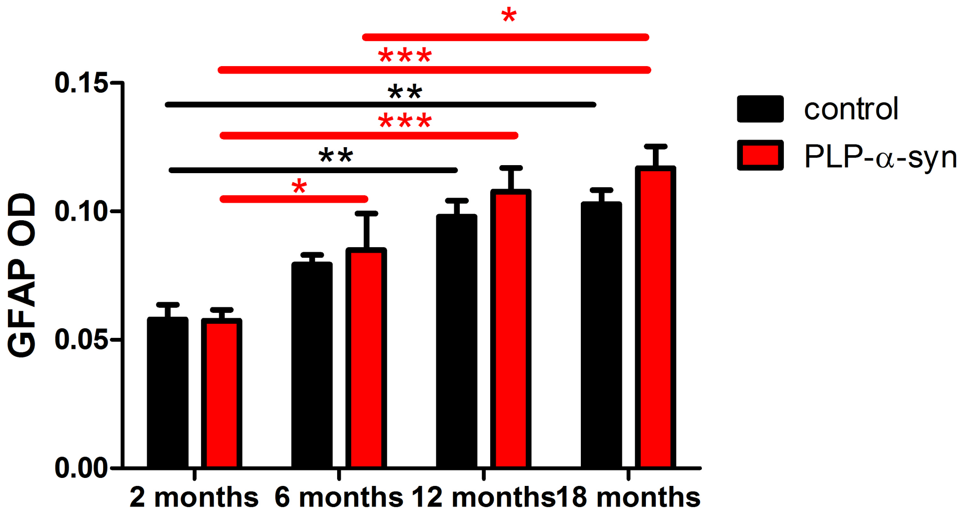

Suppl. Fig. 1. Progression of astrogliosis in control and PLP-α-syn mice was assessed by measuring GFAP OD. Although earlier initiation of astrogliosis, between 2 and 6 months of age, was seen in the transgenic mice, there was no significant difference between the age-matched MSA and control animals. However, a numeric increase of astrogliosis can be seen in the PLP-α-syn mice, suggesting that further analysis of astroglial activation may be warranted in the model in relevance to the postmortem finding in human MSA.

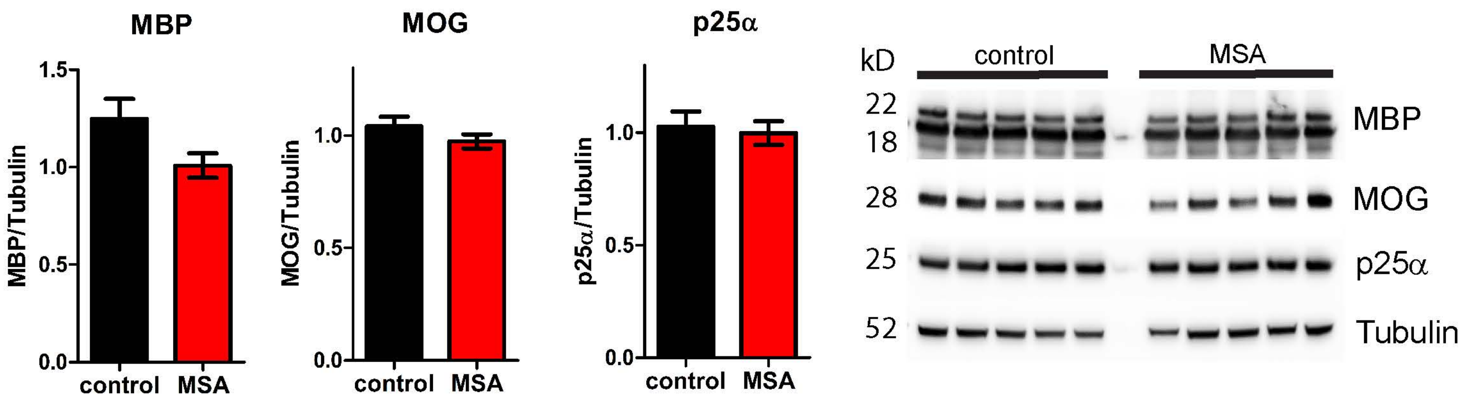

Supplementary Fig. 2. Western blot analysis does not show any major differences between control and MSA mice as of MBP, MOG and p25α protein levels at 12 months of age when the motor phenotype is detectable (unpaired t-test for MBP: $t=1.997$, $df=8$, $p=0.0809$; unpaired t-test for MOG: $t=1.252$, $df=8$, $p=0.2458$: unpaired t-test for p25α: $t=0.3305$, $df=8$, $p=0.7495$).

Supplementary Table 1. The table represents the changes in the different subtypes of microglia (A, B, C and D) in the different brain regions. Legend: ↑↑ significant increase in MSA ($p<0.016$); ↓↓ significant decrease in MSA ($p<0.016$); ns, no significant difference. Statistical analysis was done by repeated measures ANOVA with post hoc Bonferroni test with correction for multiple comparisons.

Microglia activation profile	Substantia nigra Control vs MSA		Striatum Control vs MSA		Pontine nuclei Control vs MSA		Inferior olives Control vs MSA	
2 months of age	***n=5***	***n=5***	***n=5***	***n=5***	***n=5***	***n=4***	***n=4***	***n=5***
Type A	ns		ns		ns		ns	
Type B	ns		ns		ns		ns	
Type C	ns		ns		ns		ns	
Type D	ns		ns		ns		ns	
5 months of age	***n=5***	***n=6***	***n=5***	***n=4***	***n=4***	***n=4***	***n=6***	***n=4***
Type A	↓↓		ns		ns		ns	
Type B	↑↑		ns		ns		ns	
Type C	ns		ns		ns		ns	
Type D	↑↑		ns		ns		ns	
15 months of age	***n=4***	***n=4***	***n=5***	***n=5***	***n=6***	***n=4***	***n=5***	***n=4***
Type A	↓↓		ns		ns		ns	
Type B	ns		ns		ns		ns	
Type C	↑↑		ns		ns		ns	
Type D	ns		↑↑		ns		ns	

Supplementary Table 2. The table represents the concentrations of the different cytokines and chemokines measured in the brains of control and MSA mice at 2, 5 and 15 months of age by an immunoassay. Data are presented as mean±SD.

age	2 months		5 months		15 months	
genotype	control	PLP-αSyn	control	PLP-αSyn	control	PLP-αSyn
n	4	4	4	4	4	4
TNF-α	8.63 ± 0.69	6.56 ± 0.24	8.38 ± 0.68	7.05 ± 0.32	8.59 ± 0.14	7.73 ± 0.5
ENA-78 / CXCL5	99.05 ± 19.92	103.16 ± 24.93	98.24 ± 15.34	91.12 ± 23.15	64.22 ± 3.34	72.61 ± 1.96
Eotaxin	41.44 ± 4.89	36.66 ± 2.94	40.85 ± 7.83	42.68 ± 6.61	45.8 ± 1.92	84.45 ± 72.67
G-CSF	3.37 ± 0.48	2.59 ± 0.39	3.19 ± 0.43	2.95 ± 0.66	3.17 ± 0.39	2.62 ± 0.31
GM-CSF	nd	nd	nd	nd	nd	nd
GRO-α / CXCL1	nd	nd	nd	nd	nd	nd
INF-α	nd	nd	nd	nd	nd	nd
INF-γ	6.38 ± 0.8	5.07 ± 0.22	5.7 ± 0.47	4.86 ± 0.15	5.19 ± 0.21	4.61 ± 0.3
IL-1α	9.05 ± 1.94	9.32 ± 1.78	8.55 ± 0.88	9.7 ± 1.63	7.5 ± 0.72	9.63 ± 0.49
IL-1β	31.41 ± 3.55	23.53 ± 1.17	24.85 ± 3.69	22.57 ± 1.92	20.45 ± 3.88	24.02 ± 1.33
IL-2	575.2 ± 46.69	417.74 ± 40.75	496.86 ± 49.83	446.2 ± 130.38	578.36 ± 42.01	418.48 ±2.44
IL-3	nd	nd	nd	nd	nd	nd
IL-4	3.53 ± 0.41	2.95 ± 0.3	3.27 ± 0.17	2.93 ± 0.44	3.28 ± 0.31	2.75 ± 0.15
IL-5	8.5 ± 0.85	6.97 ± 0.6	8.06 ± 1.38	7.85 ± 1.78	8.41 ± 0.66	7.21 ± 0.25
IL-6	12.65 ± 1.8	10.68 ± 0.68	11.83 ± 2.44	15.8 ± 7.49	11.2 ± 1.6	13.03 ± 1.54
IL-9	nd	nd	nd	nd	nd	nd
IL-10	54.15 ± 10.31	37.38 ± 5.13	46.01 ± 5.84	40.75 ± 5.13	49.26 ± 7.78	42.75 ± 4
IL-12p70	4.75 ± 0.72	3.82 ± 0.21	4.22 ± 0.26	3.78 ± 0.22	3.97 ± 0.13	3.54 ± 0.23
IL-13	7.67 ± 0.65	6.48 ± 0.23	6.86 ± 0.64	5.99 ± 0.25	6.58 ± 0.55	6.46 ± 0.28
IL-15	3.99 ± 0.21	3.43 ± 0.48	4.29 ± 1.03	3.78 ± 0.72	3.73 ± 0.45	3.21 ± 0.43
IL-17a	16.15 ± 3.12	10.67 ± 1.94	16.55 ± 1.84	12.73 ± 2.9	20.22 ± 7.63	14.68 ± 3.48
IL-18	173.91 ± 6.21	152.39 ± 7.2	169.17 ± 6.14	152.92 ± 7.28	165.82 ± 5.72	142.84 ± 7.23
IL-22	29.21 ± 7.83	21.89 ± 1.97	41.7 ± 15.21	20.85 ± 3.66	41.41 ± 15.3	21.54 ± 3.95
IL-23	172.91 ± 174.21	134.83 ± 28.13	137.26 ± 31.04	114.03 ± 65.19	192.79 ± 61.54	150.53 ± 8.84

age	2 months		5 months		15 months	
**genotype**	**control**	**PLP-αSyn**	**control**	**PLP-αSyn**	**control**	**PLP-αSyn**
**n**	**4**	**4**	**4**	**4**	**4**	**4**
IL-27	16.28 ± 1.34	13.81 ± 1.56	15.47 ± 0.96	13.28 ± 0.87	15.58 ± 1.1	12.54 ± 0.32
IL-28	1349.72 ± 155.24	1110.2 ± 88.6	1148.17 ± 91.55	1115.62 ± 154.4	1369.26 ± 121.6	1092.28 ± 15.7
IL-31	32.87 ± 3.12	27.93 ± 2.83	30.58 ± 0.27	25.95 ± 2.63	24.28 ± 2.24	23.96 ± 2.09
IP-10 / CXCL10	31.18 ± 8.15	35.57 ± 11.2	46.07 ± 13-74	38.7 ± 8.02	87.4 ± 26.81	89.73 ± 32.94
LIF	nd	nd	nd	nd	nd	nd
MCP-1 / CCL2	nd	nd	nd	nd	nd	nd
MCP-3 / CCL7	134.99 ± 11.06	112.77 ± 10.74	161.68 ± 103.33	117.84 ± 32.41	407.33 ± 369.98	113.94 ± 14.27
M-CSF	47.8 ± 3.3	44.18 ± 5.75	47.7 ± 15.81	59.18 ± 13.17	41.09 ± 9.7	68.41 ± 25.53
MIP-1α / CCL3	6.7 ± 1.34	9.99 ± 1	7.52 ± 2.24	15.61 ± 6.58	15.88 ± 2.45	29.45 ± 13.52
MIP-1 β	nd	nd	nd	nd	nd	nd
MIP-2 / CXCL2	11.48 ± 2	9.61 ± 0.69	10.35 ± 0.48	9.69 ± 0.77	11.6 ± 0.99	11.4 ± 0.81
RANTES / CCL5	10.77 ± 1.72	7.44 ± 1.3	11.95 ± 3.47	10.04 ± 1.03	18.98 ± 4.56	30.43 ± 19.01

Age	2 months			5 months			15 months		
**SN**									
Type A	mean	SEM	n	mean	SEM	n	mean	SEM	n
control	7024,2	458,6	5	8532,8	353,6	5	7046,75	564,4	4
MSA	8951,2	1038	5	5035,16667	514,5	6	3459,5	220,9	4
Type B									
control	4036,8	400,1	5	2941,6	807,3	5	6859,75	497	4
MSA	4312,2	488,9	5	7126,16667	666,5	6	8565,25	2046	4
Type C									
control	0	0	5	0	0	5	53,5	30,89	4
MSA	55,6	23,05	5	1072,16667	321,1	6	2913,5	914	4
Type D									
control	0	0	5	0	0	5	26,75	26,75	4
MSA	0	0	5	376,333333	118,9	6	318	99,95	4
**STRIATUM**									
Type A	mean	SEM	n	mean	SEM	n	mean	SEM	n
control	87714,8	6121	5	76105,6	3202	5	51746,8	3062	5
MSA	91142	6232	5	56910,75	1076	5	45208,6	3303	5
Type B									
control	12744,8	2581	5	25062,2	3881	5	53056,2	2584	5
MSA	19492,2	4948	5	35297,5	4510	5	53929	1427	5
Type C									
control	0	0	5	0	0	5	390,6	159,5	5
MSA	428,4	262,3	5	133,75	119,6	5	1109,2	347,3	5
Type D									
control	0	0	5	0	0	5	0	0	5
MSA	0	0	5	0	0	5	634,2	207,6	5
**PN**									
Type A	mean	SEM	n	mean	SEM	n	mean	SEM	n
control	2659,8	473,9	5	2928,25	343	4	3329,33333	336,6	6
MSA	2890	263,5	4	2247,75	207,9	4	2517,75	425,7	4
Type B									
control	70	28,82	5	112,5	48,33	4	972,166667	149,1	6
MSA	291,75	38,25	4	394,25	155,2	4	695,75	223,2	4
Type C									
control	0	0	5	0	0	4	237,333333	38,84	6
MSA	11	11	4	10,5	6,278	4	156,25	60,46	4
Type D									
control	0	0	5	0	0	4	222,5	29,14	6
MSA	16,75	16,75	4	17,5	12,03	4	134,5	56,72	4
**IO**									
Type A	mean	SEM	n	mean	SEM	n	mean	SEM	n
control	1909,5	88,57	4	2264,66667	227,6	6	3095	147,9	5
MSA	2249	132,5	4	2122,75	164,1	4	2132	170,9	4
Type B									
control	49	13,08	4	214,333333	54,05	6	988,6	67,87	5
MSA	301,8	82,02	4	166,25	50,86	4	342,75	142	4
Type C									
control	0	0	4	0	0	6	158,8	39,72	5
MSA	45,4	21,69	4	21	15,85	4	73	8,756	4
Type D									
control	0	0	4	0	0	6	176,2	42,06	5
MSA	59,6	33,9	4	5,5	5,5	4	56,25	17,97	4

Suppl. Table 3. Numbers of microglia subtypes in different brain regions. SN, substantia nigra; PN, pontine nuclei; IO, inferior olives.
